# Association of HBsAg levels with differential gene expression in NK, CD8 T, and memory B cells in treated patients with chronic HBV

**DOI:** 10.1016/j.jhepr.2023.100980

**Published:** 2023-12-03

**Authors:** Boris J.B. Beudeker, Zgjim Osmani, Gertine W. van Oord, Zwier M.A. Groothuismink, Robert J. de Knegt, Remco M. Hoogenboezem, Eric M.J. Bindels, Harmen J.G. van de Werken, Andre Boonstra

**Affiliations:** 1 Department of Gastroenterology and Hepatology, Erasmus University Medical Center, Rotterdam, the Netherlands; 2 Department of Hematology, Erasmus MC Cancer Institute, University Medical Center, Rotterdam, the Netherlands; 3 Department of Immunology, Erasmus University Medical Center, Rotterdam, the Netherlands

**Keywords:** HBsAg, hepatitis B, liver fine-needle aspirates, single-cell RNA sequencing

## Abstract

**Background & Aims:**

HBsAg secretion may impact immune responses to chronic HBV infection. Thus, therapeutic approaches to suppress HBsAg production are being investigated. Our study aims to examine the immunomodulatory effects of high and low levels of circulating HBsAg and thereby improve our understanding of anti-HBV immunity.

**Methods:**

An optimized 10x Genomics single-cell RNA sequencing workflow was applied to blood samples and liver fine-needle aspirates from 18 patients undergoing tenofovir/entecavir (NUC) treatment for chronic HBV infection. They were categorized based on their HBsAg levels: high (920-12,447 IU/ml) or low (1-100 IU/ml). Cluster frequencies, differential gene expression, and phenotypes were analyzed.

**Results:**

In the blood of HBV-infected patients on NUC, the proportion of *KLRC2*+ “adaptive” natural killer (NK) cells was significantly lower in the HBsAg-high group and, remarkably, both *KLRC2*+ NK and *KLRG1+* CD8 T cells display enrichment of lymphocyte activation-associated gene sets in the HBsAg-low group. High levels of HBsAg were associated with mild immune activation in the liver. However, no suppression of liver-resident *CXCR6+ NCAM1+* NK or *CXCR6+ CD69+* CD8 T cells was detected, while memory B cells showed signs of activation in both the blood and liver.

**Conclusions:**

Among NUC-treated patients, we observed a minimal impact of HBsAg on leukocyte populations in the blood and liver. However, for the first time, we found that HBsAg has distinct effects, restricted to NK-, CD8 T-, and memory B-cell subsets, in the blood and liver. Our findings are highly relevant for current clinical studies evaluating treatment strategies aimed at suppressing HBsAg production and reinvigorating immunity to HBV.

**Impact and implications:**

This study provides unique insight into the impact of HBsAg on gene expression levels of immune cell subsets in the blood and liver, particularly in the context of NUC-treated chronic HBV infection. It holds significant relevance for current and future clinical studies evaluating treatment strategies aimed at suppressing HBsAg production and reinvigorating immunity to HBV. Our findings raise questions about the effectiveness of such treatment strategies and challenge the previously hypothesized immunomodulatory effects of HBsAg on immune responses against HBV.

## Highlights

• HBsAg has distinct effects in the blood and liver of patients with chronic HBV.
• The effects of HBsAg are restricted to NK-, CD8 T-, and memory B-cell subsets.
• High HBsAg levels are associated with mild immune activation, but not suppression, of these immune cell subsets in the liver.

## Graphical abstract

**Figure:**
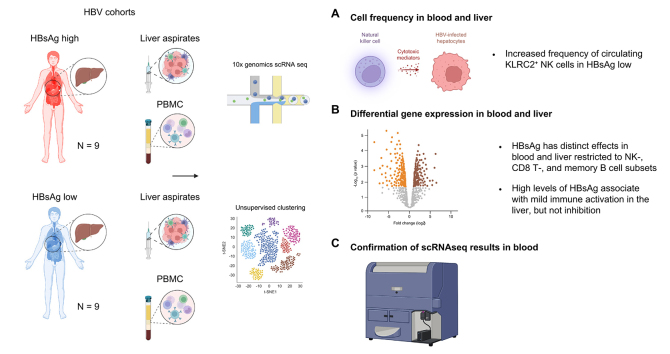

## Introduction

Chronic HBV infection affects over 300 million people worldwide and is one of the most common risk factors for the development of cirrhosis and hepatocellular carcinoma.^1^ Although nucleos(t)ide analogue (NUC) treatment can lead to long-term control of viremia, curative therapy for chronic HBV infection is still lacking. It is well known that persistent viral infection and prolonged exposure to high levels of viral antigens negatively impact virus-specific T-cell responses, as shown in lymphocytic choriomeningitis virus-infected mouse studies.^2^ In line with this, it has been postulated that continuous secretion of non-infectious HBsAg by infected hepatocytes is immunomodulatory and may induce T-cell exhaustion, thereby contributing to a weak immune response against HBV, as observed in chronically infected patients.[3], [4], [5], [6], [7] Various siRNA-based therapeutic approaches aimed at suppressing HBsAg production from both integrated HBV DNA and covalently closed circular DNA are currently being investigated in clinical trials.^8^ However, the potential benefits of such treatments on immune responses to HBV are not clearly understood.

Previously, we evaluated the effects of the levels of circulating HBsAg on peripheral blood leukocytes of patients with chronic HBV using fluorescence-activated cell sorting and microarray analysis.^9^ We observed minor differences in gene expression profiles of purified leukocyte subpopulations. Other studies have confirmed that HBsAg has minimal impact on cellular immune responses. This has been demonstrated in mouse models of HBV infection^10^ and in the blood of patients with chronic HBV.^7^ However, *in vivo* animal models for HBV do not completely mimic the chronicity of host-pathogen interactions in humans. Previous attempts only evaluated the effects of HBsAg on leukocytes in the blood and did not examine the consequence in the liver. Therefore, we previously assessed the intrahepatic transcriptome of patients with chronic HBV and differential HBsAg levels by bulk RNA sequencing of liver biopsies.^11^ We observed that HBsAg had minimal impact on the liver transcriptome, although genes involved in leukocyte activation, recruitment, and innate responses were positively correlated with liver HBsAg levels. It is unclear if the correlations between HBsAg levels and gene expression patterns in the liver indicate HBsAg-related immune activation due to the limitations of bulk RNA sequencing. Our study aims to address a significant gap in knowledge regarding the putative immunomodulatory effects of HBsAg. By examining blood and liver fine-needle aspirates (FNAs) with single-cell RNA sequencing (scRNAseq), we hope to shed light on the impact of high and low circulating HBsAg levels on immune cell subsets. Unbiased bioinformatic analyses provided us with unique insights into the effects of HBsAg levels. We are the first to show that: (1) HBsAg levels have distinct effects in the blood and liver of patients with chronic HBV, (2) the effects of HBsAg levels are restricted to natural killer (NK)-, CD8 T-, and memory B-cell subsets, (3) and that high HBsAg levels are associated with mild immune activation, but not suppression, in the liver.

## Materials and methods

### Study population

A total of 19 patients with HBeAg-negative chronic hepatitis B visiting the outpatient clinic of the Erasmus MC were enrolled in this study. Included patients were at least 18 years of age, received NUC treatment (*i.e*., entecavir or tenofovir) and had undetectable viral load (HBV DNA <20 IU/ml). Clinical data were collected from electronic medical records. Serum levels of HBsAg (IU/ml) were measured using the Lumipulse G HBsAg assay (Fujirebio Europe) on a LUMIPULSE G1200 analyzer (Fujirebio Inc). Patients with relatively low (≤100 IU/ml) and high serum HBsAg levels (>900 IU/ml) were selected for inclusion. Liver fibrosis was scored using liver elastography (Fibroscan®, Echosens, France) or liver pathology. Patients were excluded in case of significant liver fibrosis (elastography >7.0 kPa or metavir >F1), history of hepatic decompensation, history of hepatocellular carcinoma, co-infection with HCV, HDV, HEV or HIV, presence of auto-immune liver disease, severe liver steatosis, steatohepatitis, hemochromatosis, Wilson’s disease, documented clinical history of alcohol abuse, malignancies, or recent pregnancy.

### Collection of blood and liver FNAs

Heparinized blood samples were collected from patients for isolation of peripheral blood mononuclear cells (PBMCs) using ficoll separation (Ficoll-Paque™ plus, GE Healthcare Bio-Sciences AB). PBMCs were immediately processed fresh or were stored for up to 24 h at -80 °C and subsequently frozen at -150 °C for long-term storage. FNAs of the liver were collected from nine patients. To minimize confounders (*i.e*., blood artifacts or ischemic cell death) a rapid aspirate-processing pipeline was developed to collect intrahepatic leukocytes. FNAs were freshly collected at the outpatient clinic and immediately transferred on ice and processed for scRNAseq. A workflow time of under 1 hour was established from the collection of liver aspirates to single-cell droplet encapsulation, as described previously.^12^ Briefly, for each patient four ultrasound-guided passes of intrahepatic aspirates were collected using a 25-gauge spinocan® needle. Passes were collected in 500 μl colorless RPMI and immediately stored on ice for transport. Optical density measurement (OD: 415-595 nm) was applied to identify samples with blood contamination. Samples with an OD value of ≥0.19 were considered contaminated with blood and were therefore excluded. Samples that passed quality control were pooled and the remaining red blood cells were removed by 5 min incubation with Red Blood Cell Removal (Stemcell). Cells were washed twice and resuspended in RPMI + 5% FCS (700-1200 cells/μl) for further processing of fresh FNAs.

### Sample preparation and 10x genomics scRNAseq

PBMCs were prepared and sequenced in two batches, and FNAs were processed in one batch. FNAs and the first batch of PBMCs were prepared according to the 10x Genomics Single Cell 3’ v3 Reagent Kit user guide. The second batch of PBMCs were prepared according to the 10x Genomics Single Cell 5’ v2 Reagent Kit user guide. Briefly, the maximum volume was loaded on a 10x Genomics Chromium Controller in order to aim for a maximum recovery rate of 10,000 cells. After droplet generation, samples were transferred into a pre-chilled tube strip and cDNA was generated. As outlined by the user guide, cDNA was recovered using Recovery Agent provided by 10x Genomics, and subsequently purified using a Silane DynaBead mix (Thermo Fisher). Purified cDNA was amplified for 13 cycles before being purified again using SPRIselect beads (Beckman Coulter). cDNA concentrations of the samples were determined on a Bioanalyzer (Agilent Technologies). Libraries were prepared as outlined by the 10x Genomics Single Cell 3’ v3 or 5’ v2 Reagent Kit user guide and sequenced (28-8-0-91 cycles) on a NovaSeq6000 platform (Illumina, single-end 90 base pair reads).

### Quality control and filtering of scRNAseq data

To process raw files to count matrices, 10x Genomics Cell Ranger 6.1.2 with the human reference genome GRCh38 was used with default settings. Cell Ranger-processed filtered feature matrices were analyzed using R version 4.2.2 and Seurat version 4.3.0. Sequencing data from PBMCs were obtained in two batches, data files were merged, log-normalized and data integration was applied using reciprocal principal component analysis (k.anchor = 20, dims = 50) to correct for batch effects. Data from FNAs were obtained in one batch and were merged and analyzed separately from blood, hence data integration of blood with FNAs was not applied. Low-quality cells or empty droplets were filtered by removing cells with <600 features and >20% of reads mapped to the mitochondrial genome. Cell doublets or multiplets were removed by excluding cells with >4,000 features. Out of 19 blood samples and 9 FNAs, one liver sample did not pass quality control and was excluded due to low-quality cells as described by the criteria above. Single-cell clustering was performed on the remaining 19 blood and 8 paired liver samples from 19 enrolled patients. Since our study intended to study patients on long-term NUC treatment, one blood sample from a patient that only received NUC treatment for a period of 3 months was included in the clustering (PBMC11) but subsequently removed from further downstream analysis. We continued the analysis with 18 blood samples and 8 paired liver samples.

### Analysis of scRNAseq data

For the regular single-cell analysis, the default settings from the Seurat pipeline in R were used for normalization, highly variable gene selection, dimensionality reduction, and clustering. Cells from both PBMCs (n = 18) and FNAs (n = 8) were clustered separately using the Louvain algorithm (dims = 20, resolution = 1.0) and clusters were visualized by uniform manifold approximation and projection (dims = 1:20, n.neighbors = 15). Cell clusters were annotated manually, based on well-known lineage-specific markers retrieved from differentially expressed genes (DEGs) using the FindAllMarkers function (min.pct = 0.15, logfc.threshold = 0.6, only.pos = TRUE), including genes that are expressed in at least 15% of cells and with a significant absolute fold-change of 1.5 (*p <*0.05). Small clusters with less than 500 cells were excluded from further downstream DEG analysis. DEG analysis was performed to compare the single-cell transcriptome of each cluster between patients with high *vs.* low HBsAg serum levels using the FindMarkers function. To assign significant DEGs, genes that were expressed by at least 10% of cells in one of both groups were included and a cut-off value of 1.5 for absolute fold-change and *p*-adjusted <0.05 was applied. *P* values were obtained using the Wilcoxon rank-sum test and adjusted using the Benjamini Hochberg false discovery rate correction method.

### Gene set-enrichment analysis

Gene expression analyses between the HBsAg-high and low groups were assessed for overrepresentation of gene sets related to biological states or processes. Results were analyzed as a ranked list of genes, sorted by fold changes in decreasing order. A normalized enrichment score was calculated for each gene set, reflecting the degree to which it is overrepresented at the top or bottom of the ranked list. *P* values were calculated based on permutations using the R-package clusterProfiler and were corrected with the Benjamini Hochberg false discovery rate correction method.

### FACS validation

Frozen PBMCs were thawed and washed with RPMI 1640 supplemented with 10% FCS (Lonza, Walkersville, MD, USA). For flow cytometry, 500,000 viable PBMCs were incubated for 20 minutes at 4 °C in the dark with the desired mixture of antibodies (supplementary CTAT table). Data was acquired with the FACS Canto II or FACS Symphony analyzer and analyzed using FlowJo version 10.9.0 (Tree Star Inc.).

### Statistical analyses

R (version 4.2.2) and GraphPad Prism (version 8) were used for statistical analyses. Continuous variables were expressed as median (IQR). The Wilcoxon rank-sum test or χ^2^-squared test in the analysis of contingency tables was applied when appropriate. Results were considered statistically significant if the adjusted *p* value was <0.05.

### Ethics and funding

This study was conducted according to the guidelines of the Declaration of Helsinki and the principles of Good Clinical Practice. The ethical review board of the Erasmus MC approved the study, registered as MEC-2008-146 and MEC-2010-039. We obtained written informed consent from patients prior to inclusion. The Foundation for Liver and Gastrointestinal Research (SLO) sponsored the study. The funding source did not influence the study design, data collection, analysis and interpretation of the data, writing of the report, or the decision to submit for publication.

## Results

### Patient characteristics

A total of 18 patients with a stably suppressed viral load (undetectable [<20 IU/ml] HBV DNA) were included in the study (Table 1). All patients were HBeAg-negative (<0.01 PEIU/ml HBeAg), had no significant fibrosis (F0/F1 stage), and received NUC therapy with a median treatment duration of 7 years. Patients were divided into two groups based on circulating HBsAg serum levels: the HBsAg-high (median 4,600 [5,795] IU/ml) and the HBsAg-low (70 [40] IU/ml) groups.

**Table 1:** Patient characteristics.

	Unit	Total	High	Low	*p* value
Patients	N	18	9	9	
Age (years)	Median (IQR)	51 (14)	48 (16)	52 (10)	0.6508
Sex (male)	N, %	17 (94%)	9 (100%)	8 (89%)	0.3035
HBeAg negative	N, %	18 (100%)	9 (100%)	9 (100%)	
Log HBV DNA (IU/ml)	Median (IQR)	Undetectable (<20)	Undetectable (<20)	Undetectable (<20)	
ALT (U/L)	Median (IQR)	24 (16)	21 (34)	26 (10)	0.4229
HBsAg (IU/ml)	Median (IQR)	510 (4529)	4,600 (5795)	70 (40)	<0.0001
Treatment regimen	N, %				0.3428
TDF		10 (56%)	6 (67%)	4 (44%)	
ETV		8 (44%)	3 (33%)	5 (56%)	
Treatment duration (years)	Median (IQR)	7 (7)	5 (3)	9 (4)	0.2854
F0/F1 fibrosis stage	N, %	18 (100%)	9 (100%)	9 (100%)	
Anti-CMV IgG	+/-/ND	13/4/1	6/3/0	7/1/1	0.3540
Anti-HBe	+/-/ND	18/0/0	9/0/0	9/0/0	
Anti-HBs	+/-/ND	0/18/0	0/9/0	0/9/0	
HBcrAg (logU/ml)	Median (IQR)	0.45 (7.85)	1.3 (8.90)	0.10 (2.18)	0.207

ALT, alanine aminotransferase; CMV, cytomegalovirus; ETV, entecavir; HBcrAg, hepatitis B core-related antigen; ND, not determined; TDF, tenofovir disoproxil fumarate.

### Low circulating HBsAg is associated with significant gene expression changes and enrichment in adaptive *KLR2C+ NCAM1*^low^ NK cells

**Fig. 1:**
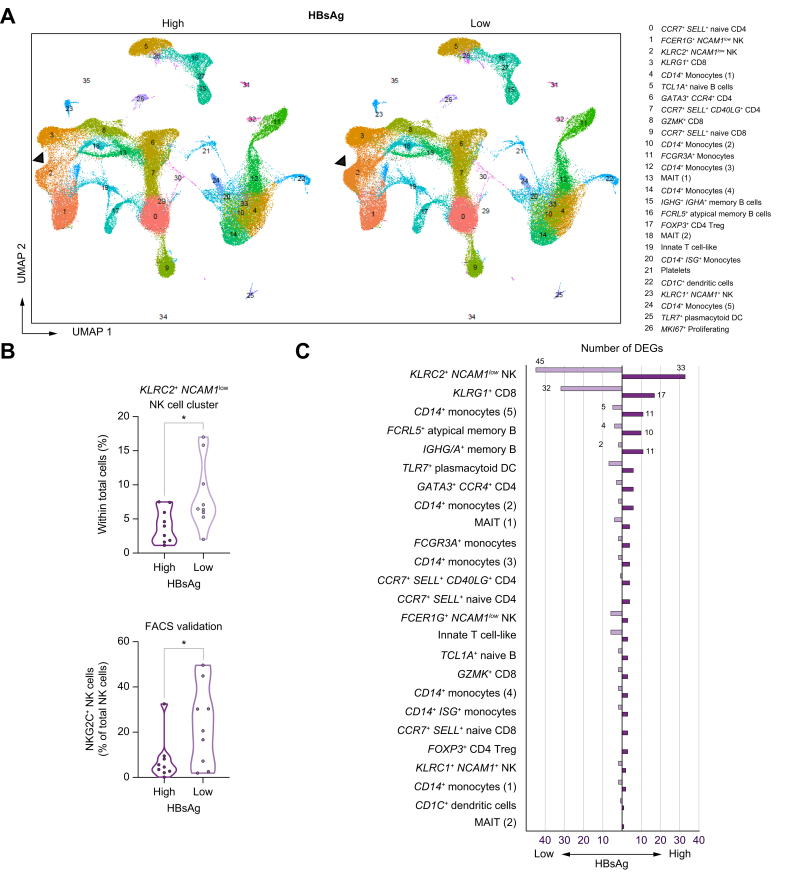
PBMC clusters. (A) UMAP scRNA clustering of PBMCs split by HBsAg level. (B) Bar plot showing the fraction of each cluster within both patient groups. The proportion of *KLRC2*+ *NCAM1*^low^ NK cells is significantly lower in the HBsAg-high group than the HBsAg-low group (*p =* 0.0053, Wilcoxon rank-sum). Flow cytometric analysis confirmed a significantly lower frequency of NKG2C+ NK cells in patients with high HBsAg levels (*p =* 0.04). (C) The total number of DEGs observed in each cluster within the HBsAg-high and HBsAg-low group. Significant changes in the expression of ribosomal and mitochondrial genes were discarded. To determine significant differences, Wilcoxon rank-sum test with FDR correction was used (adjusted *p <*0.05). DEGs, differentially expressed genes; FDR, false discovery rate; NK, natural killer; PBMCs, peripheral blood mononuclear cells; scRNA, single-cell RNA; UMAP, uniform manifold approximation and projection.

It has been postulated that continuous secretion of HBsAg by HBV is immunomodulatory and may contribute to a weak immune response against HBV, as observed in patients with chronic HBV.^5^ However, studies have shown minimal impact of HBsAg on circulating leukocyte subpopulations.^7^^,^^9^ To assess the putative immunomodulatory effects of HBsAg, we compared cell frequencies and gene expression patterns of cell clusters in blood between patients with high *vs.* low HBsAg. Clustering of 166,351 PBMCs identified 27 distinct cell clusters in peripheral blood with >500 cells (Supplementary data 1). There were no unique cell clusters observed in patients with either high or low HBsAg levels (Fig. 1A). Cluster frequencies in peripheral blood were comparable between both groups, except for the *KLRC2*+ *NCAM1*^low^ NK-cell cluster which was significantly lower in patients with high HBsAg levels (*p =* 0.0053, Fig. 1B and Fig. S1, Table S3). This cluster exhibited negative expression of *FCER1G* (FcεRγ) and high expression of *KLR2C* (NKG2C), resembling the "memory-like" NK-cell population known as NKG2C+ "adaptive" NK cells.^13^ Flow cytometry confirmed the significantly lower frequency of NKG2C+ CD56^dim^ NK cells in the blood of patients with high HBsAg levels compared to those with low HBsAg levels (4.46% (IQR 5.92) *vs.* 20.6% (IQR 23.1); *p =* 0.040) (Fig. 1B and Fig. S2, Table S4). Previously, Oliviero *et al.* reported an increased proportion of NKG2C+ NK cells in patients with chronic HBV compared to healthy controls, 11-23% *vs.* 5-13%, respectively.^14^ However, it is important to note that this comparison was in the context of chronic HBV *vs.* no infection, rather than between chronic HBV with high *vs.* low circulating levels of HBsAg. Additionally, upon subgroup analysis, we found that the *KLRC2*+ *NCAM1*^low^ NK-cell cluster was predominantly observed in patients with a documented history of human cytomegalovirus (CMV) infection (Fig. S3A). Importantly, uniform manifold approximation and projection analysis of only the patients with a history of CMV infection demonstrated a significantly lower frequency of this cluster in individuals with high HBsAg levels, which was further confirmed by flow cytometry (Fig. S3B).

**Fig. 2:**
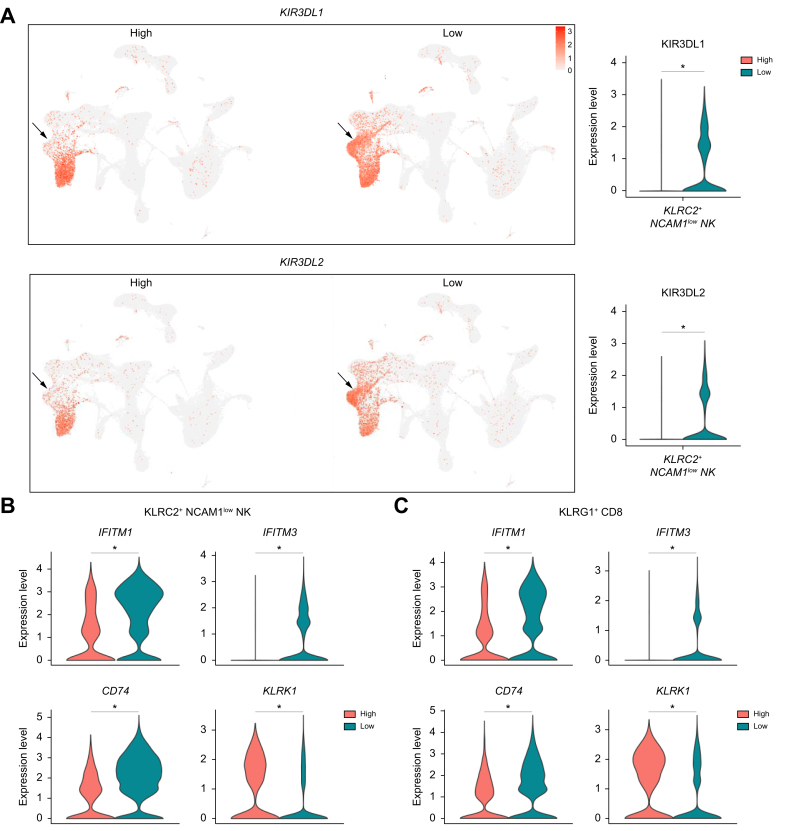
Significant gene expression changes associated with HBsAg in adaptive *KLR2C*+ *NCAM1*^low^ NK- and *KLRG1*+ CD8 T-cell clusters. (A) Feature plot split by HBsAg level showing relative log gene expression levels of *KIR3DL1* and *KIR3DL2*. Each dot represents a single cell, relative log gene expression levels are represented by a color scale and violin plot on the right side of the figure. The black arrow points towards the *KLRC2*+ *NCAM1*^low^ NK-cell cluster. Gene expression levels of *IFITM1*, *IFITM3*, *CD74* and *KLRK1* are shown in violin plots for the (B) *KLRC2*+ *NCAM1*^low^ NK-cell and (C) *KLRG1+* CD8 T-cell clusters. For determining significant differences, Wilcoxon rank-sum test with FDR correction was used (adjusted *p <*0.05). FDR, false discovery rate; NK, natural killer.

**Fig. 3:**
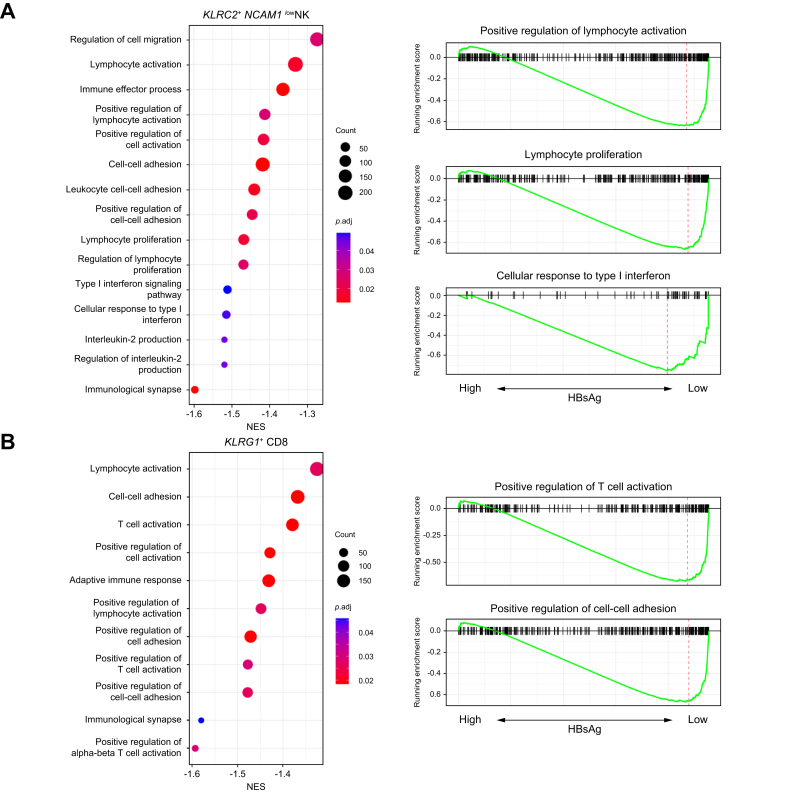
GSEA of gene sets involved in lymphocyte-/T-cell activation in *KLR2C+ NCAM1*^low^ NK and *KLRG1*+ CD8 T-cell clusters. GSEA dot plots showing gene sets and corresponding NES that are significantly enriched by the *KLRC2*+ *NCAM1*^low^ NK-cell (A) and *KLRG1+* CD8 T-cell cluster (B) within the HBsAg-low group compared to the HBsAg-high group. Gene sets with negative NES are enriched in the HBsAg-low group. Color and size of dots represents the adjusted *p* values and gene count, respectively. On the right side of the figure, GSEA plots are shown for a selection of gene sets. The vertical black lines in the running enrichment score (green line) show where the members of the gene set appear in the ranked list of genes. The red dotted line represents the maximum enrichment score. *P* values were corrected with FDR correction method (adjusted *p <*0.05). FDR, false discovery rate; GSEA, gene set-enrichment analysis; NES, normalized enrichment scores.

DEG analysis was performed to compare the individual clusters within patients with high *vs.* low HBsAg levels (Table S5). As shown in Fig. 1C, the highest number of DEGs in blood were observed in the *KLRC2*+ *NCAM1*^low^ NK-cell cluster, followed by the *KLRG1+* CD8 T-cell cluster, one *CD14+* monocyte cluster, and the *FCRL5+* atypical memory B-cell and *IGHG/A+* memory B-cell clusters. Out of the 45 DEGs, inhibitory killer-cell immunoglobulin-like receptors (KIRs) were highly expressed by the *KLRC2*+ *NCAM1*^low^ NK-cell cluster within the HBsAg-low group (Fig. 2A). Previously, predominant expression of inhibitory KIRs has been described in NKG2C+ NK cells in patients with chronic HBV.^15^ We show that the expression of these inhibitory KIRs is associated with low HBsAg levels. Among others, expression of interferon-related genes (*IFITM1*, *IFITM3*) and *CD74* (MIF receptor) was significantly higher within the HBsAg-low group in both the *KLRC2*+ *NCAM1*^low^ NK and *KLRG1*+ CD8 T-cell cluster (Fig. 2B,C and Fig. S4). Whereas the activating *KLRK1* (NKG2D) receptor was highly expressed within the HBsAg-high group in both clusters (Fig. 2B,C). Interestingly, both clusters shared similar gene expression changes, 42 out of 49 DGEs in the *KLRG1+* CD8 T-cell cluster overlapped with DEGs of the *KLRC2*+ *NCAM1*^low^ NK-cell cluster. This overlap was confirmed by gene set-enrichment analyses in which gene sets involved in lymphocyte-/T-cell activation and cell-cell adhesion were significantly enriched in both clusters in the HBsAg-low group compared to the HBsAg-high group (Fig. 3, Table S6). However, gene sets involved in the type I interferon response, IL-2 production, and lymphocyte proliferation were only significantly enriched by the *KLRC2*+ *NCAM1*^low^ NK-cell cluster within the HBsAg-low group. Our data reveal that the frequency of *KLRC2*+ *NCAM1*^low^ “adaptive” NK cells is lower in the blood of patients with relatively high HBsAg levels, and at the same time these cells display gene expression patterns reflective of an activated state with significant enrichment of proliferation-associated gene sets in the HBsAg-low group.

Two memory B-cell clusters were also found in the top five cell types primarily affected by HBsAg (Fig. 1C). Both *CD83* and *CXCR4* expression was significantly higher in these clusters within the HBsAg-high group compared to the HBsAg-low group (Fig. S5). CD83 has been identified as an activation marker for B cells and previous studies have shown an activating gene signature in B cells of patients with chronic HBV.[16], [17], [18], [19], [20] In addition, CXCR4 was shown to be upregulated by B cells in chronic HBV when compared to healthy controls.^16^^,^^18^ Herein, we show that higher *CD83* and *CXCR4* gene expression by memory B cells in the blood of patients with chronic HBV is significantly associated with high HBsAg levels.

To note, the highest number of DEGs were observed in the *MKI67+* proliferating cell cluster. However, this cluster had a relatively low number of cells (n = 850) and consisted of multiple proliferating cell types. Subcluster analysis was underpowered due to low absolute cell counts and therefore the *MKI67+* proliferating cell cluster was not assessed further. Interestingly, for many clusters, relatively high HBsAg levels were associated with higher expression of genes involved in AP-1 and NF-κB signaling (*e.g*., *JUNB*, *FOS*, *NFKBIA*, and *TNFAIP3*).

In sum, our findings demonstrate for the first time a significant decrease in the proportion of *KLRC2*+ *NCAM1*^low^ "adaptive" NK cells in the peripheral blood of patients with high HBsAg levels, and this was validated at the protein level using flow cytometry. Additionally, we observed a notable enrichment of gene sets associated with lymphocyte activation in both the *KLRC2*+ *NCAM1*^low^ NK-cell and *KLRG1+* CD8 T-cell clusters in the HBsAg-low group.

### High HBsAg is associated with minor gene expression changes in *CXCR6+ CD69+* liver-resident CD8 T cells, pointing towards a more activated cell state

**Fig. 4:**
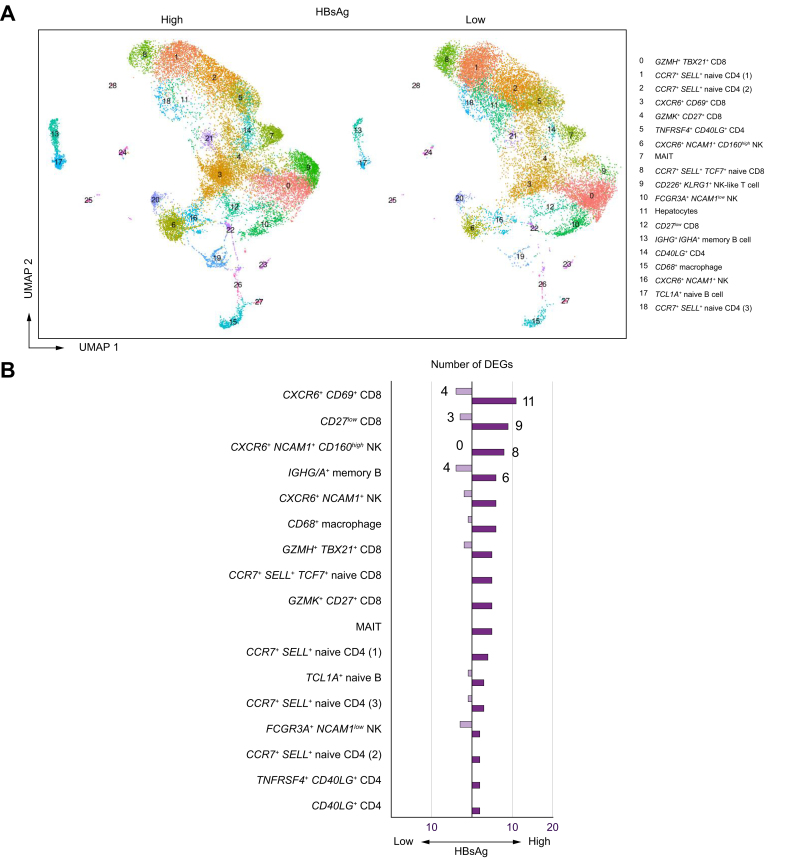
Clustering of liver immune subsets and DEGs between the HBsAg-high and -low group. (A) UMAP scRNA clustering of immune subsets in FNAs split by HBsAg level. (B) The total number of DEGs observed in each cluster within the HBsAg-high and HBsAg-low groups. Significant changes in the expression of ribosomal and mitochondrial genes were discarded. To determine significant differences, Wilcoxon rank-sum test with FDR correction was used (adjusted *p <*0.05). DEGs, differentially expressed genes; FDR, false discovery rate; FNAs, fine-needle aspirates; scRNA, single-cell RNA; UMAP, uniform manifold approximation and projection.

Previously, we evaluated the effects of HBsAg in the liver by bulk RNA sequencing and found gene correlations with HBsAg that were associated with leukocyte activation.^11^ These results contradict the hypothesis that HBsAg has an immunomodulatory effect. To assess the putative immunomodulatory effects of HBsAg and to confirm our previous conflicting findings in the liver, we identified immune subsets in the liver by scRNAseq and compared frequencies and gene expression patterns of these clusters between patients with high *vs.* low HBsAg. Clustering of 35,513 intrahepatic cells identified 19 cell clusters with >500 cells (Supplementary data 2, Table S9). There were no unique cell clusters observed in patients with either high or low HBsAg levels (Fig. 4A). Immune cell cluster frequencies in liver FNAs varied between samples but were not significantly different between groups (Table S10, Fig. S6).

**Fig. 5:**
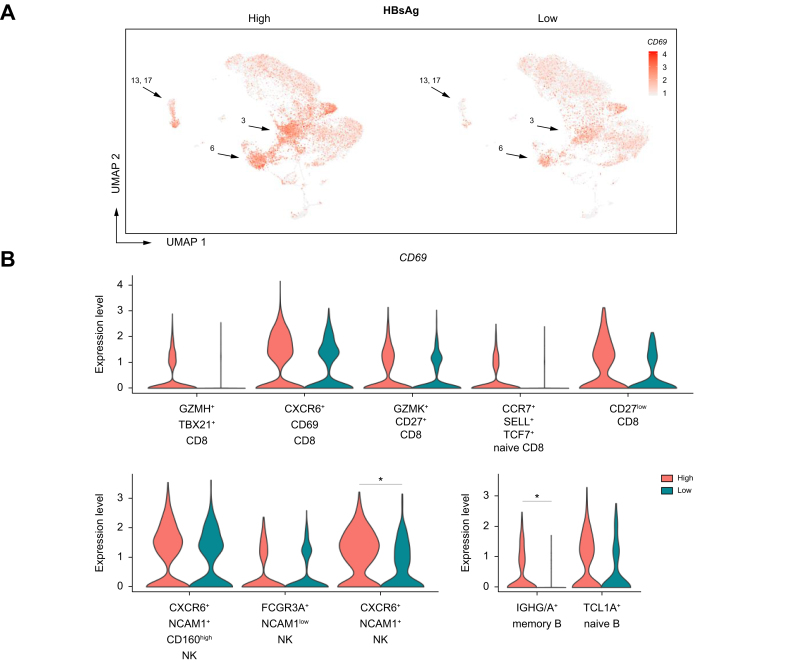
*CD69* gene expression by HBsAg level and immune cell cluster. (A) Feature plot split by HBsAg level showing relative log gene expression levels of *CD69*. Each dot represents a single cell, relative log gene expression levels are represented by a color scale. The black arrows point towards the *CXCR6+ CD69+* liver-resident CD8 T-cell cluster (3), the *CXCR6+ NCAM1+ CD160*^high^ NK-cell cluster (6), and the naive and memory B-cell cluster (13, 17). (B) *CD69* gene expression levels are shown in violin plots for all CD8-, NK- and B-cell clusters identified in the liver. For determining significant differences, Wilcoxon rank-sum test with FDR correction was used (adjusted *p <*0.05). FDR, false discovery rate; NK, natural killer.

DEG analysis was performed to compare the individual clusters within patients with high *vs.* low HBsAg levels (Fig. 4B, Table S11). In general, the number of DEGs was relatively low for clusters in liver FNAs. The highest number of DEGs were observed in the *CXCR6+ CD69+* liver-resident CD8 T-cell cluster with 15 DEGs, followed by the *CD27*^low^ CD8 T-cell, *IGHG/A+* memory B-cell, and the *CXCR6+ NCAM1+ CD160*^high^ liver-resident NK-cell cluster. Within the HBsAg-high group, the early activation marker *CD69* was found to be highly expressed in memory B-cell, NK- and CD8 T-cell clusters when compared to the HBsAg-low group in the liver (Fig. 5). In line with the observations in peripheral blood, high HBsAg levels were associated with a significantly higher expression of *CXCR4* by B-cell clusters in the liver (Fig. S7). Our findings show that memory B-cell gene expression profiles in patients with chronic HBV with relatively high HBsAg levels exhibit a more activated phenotype in both peripheral blood and liver FNAs, with a simultaneous increase in *CXCR4* expression. Studies have demonstrated the role of CXCR4 and its ligand in the recruitment of T cells to the liver in the case of hepatitis;^21^ however, the role of CXCR4 in B-cell activation and/or trafficking to the liver remains unknown. The *CXCR6+ NCAM1+ CD160*^high^ liver-resident NK-cell cluster showed a significantly higher expression of *IL32* within the HBsAg-high group. Six isoforms of IL-32 exist and are known to primarily induce various pro-inflammatory cytokines and chemokines (especially the β and γ isoforms).^22^ In hepatocytes, intracellular IL-32γ is induced by IFN-γ and TNFα, and it has been shown to suppress HBV replication^23^ and thus play a role in the non-cytopathic antiviral response. However, IL-32 production by NK cells and its exact role in the antiviral immune response against HBV remain unknown.

**Fig. 6:**
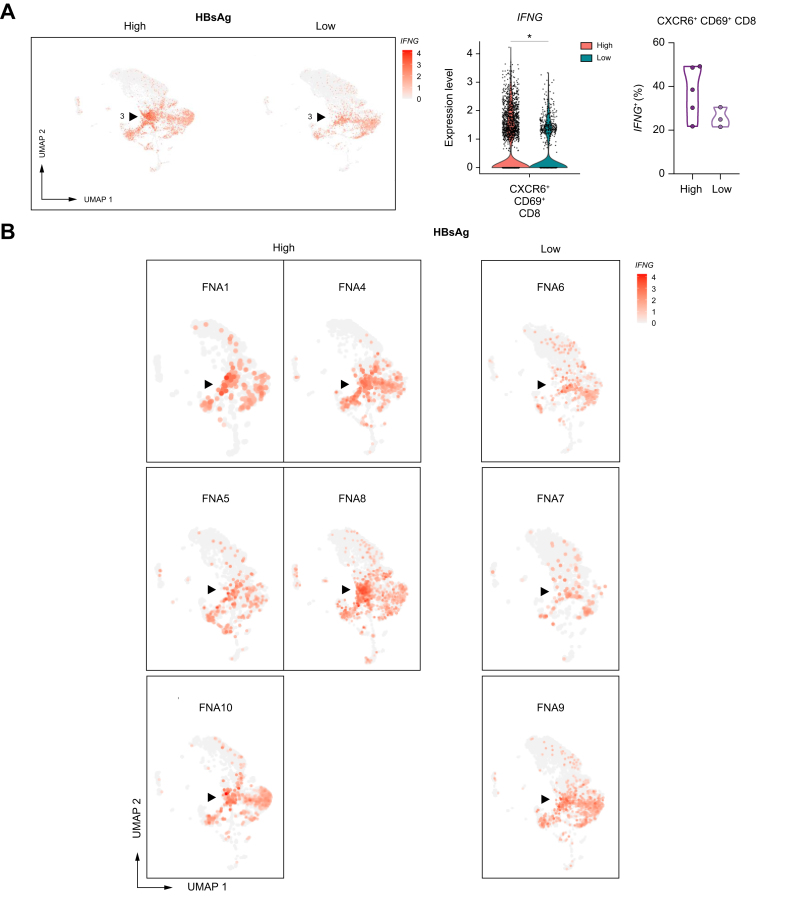
Relative log gene expression levels of *IFNG* by HBsAg levels. Each dot represents a single cell, relative log gene expression levels are represented by a color scale. The black arrows point towards the *CXCR6+ CD69+* liver-resident CD8 T-cell cluster (3). (A) Feature plot split by HBsAg level showing relative log gene expression levels of *IFNG*. On the right side of the figure, both the expression of *IFNG* and the percentage of *CXCR6+ CD69+* liver-resident CD8 T cells that express *IFNG* are shown in violin plots. To determine significant differences, Wilcoxon rank-sum test with FDR correction was used (adjusted *p <*0.05). (B) Feature plots split by individual FNAs of patients showing relative log gene expression levels of *IFNG*. FDR, false discovery rate; FNAs, fine-needle aspirates.

**Fig. 7:**
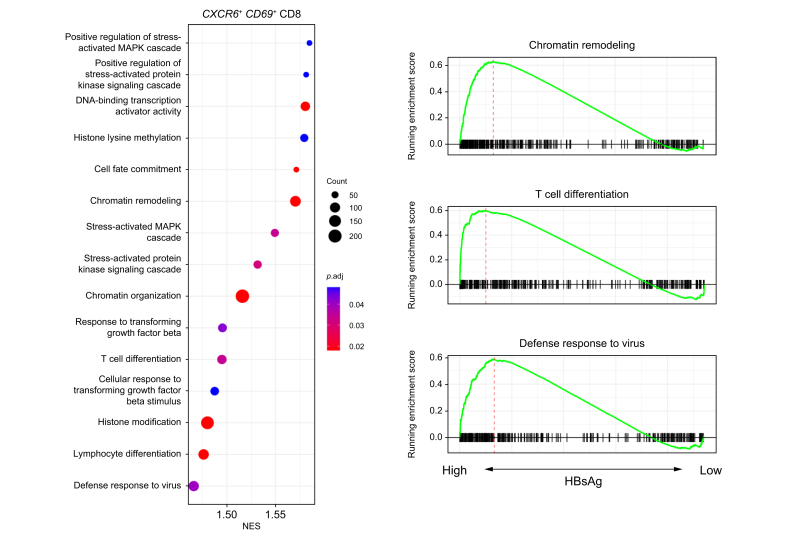
GSEA dot plot showing gene sets that are significantly enriched by the *CXCR6*+ *CD69*+ liver-resident CD8 T-cell cluster within the HBsAg-high *vs.* the HBsAg-low group. Gene sets with positive NES are enriched in the HBsAg-high group. The color and size of the dots represent the adjusted p values and gene counts, respectively. On the right side of the figure, GSEA plots are shown for a selection of gene sets. *P* values were corrected with FDR correction method (adjusted *p <*0.05). FDR, false discovery rate; GSEA, gene set-enrichment analysis.

Unique to the *CXCR6+ CD69+* liver-resident CD8 T-cell cluster was the significantly higher expression of *KLRB1* (CD161), *KLRC1* (NKG2A), and more importantly *IFNG* within the HBsAg-high group (Fig. 6). Notably, the percentage of cells within this cluster that expressed *IFNG* and *TNF* (Fig. S8) was slightly higher in the HBsAg-high group than the HBsAg-low group, albeit not significant. According to the literature, CD161-expressing CD8 T cells have tissue-homing properties and enhanced cytotoxic characteristics.^24^ CD161+ CD8 T cells secrete high levels of IFN-γ and TNF, and they have been shown to be enriched in the liver in response to chronic HCV infection.^25^ In contrast, NKG2A has been classified as a late inhibitory receptor on CD8 T cells that is expressed after repeated antigen stimulation.^26^ Interestingly, gene sets involved in chromatin remodeling, T-cell differentiation, and antiviral immune response were significantly enriched by *CXCR6+ CD69+* liver-resident CD8 T cells within the HBsAg-high group (Fig. 7, Table S12). Our results indicate that the *CXCR6+ CD69+* CD8 T cells in the HBsAg-high group exhibit a more differentiated state, as evidenced by changes in gene expression profiles associated with activation and an effector phenotype. However, we did not observe any significant differences in the frequency of the liver immune landscape, or correlations with *CXCR6+ NCAM1+* NK- and *CXCR6+ CD69+* CD8 T cells. Overall, we observed minor gene expression changes in the liver associated with differential HBsAg levels. Similar to blood, high HBsAg levels were associated with a higher expression of AP-1 signaling pathway-related genes in intrahepatic immune cell subsets (*e.g*., *FOS*, *FOSB*, *JUN*). Our data reveals that high HBsAg levels are associated with gene expression changes pointing towards mild immune activation, but not suppression, of liver-resident *CXCR6+ NCAM1+* NK- and *CXCR6+ CD69+* CD8 T cells, while memory B cells showed signs of activation in both the blood and liver of patients with high HBsAg levels.

## Discussion

Understanding the immune cell subsets crucial for mediating HBV resistance is vital to develop effective therapies and improve treatment outcomes. While targeting the HBV envelope protein holds promise, the impact of HBsAg on the immune system remains poorly understood. Utilizing unique blood and liver samples from NUC-treated patients with chronic HBV and high or low HBsAg levels, our findings suggest that the immunologic impact of HBsAg is not uniform across all leukocyte subpopulations. Instead, it appears to be restricted to NK-, CD8 T-, and memory B-cell subsets, and is suggestive of mild immune activation in those with high HBsAg levels.

We show that the frequency of *KLRC2*+ *NCAM1*^low^ “adaptive” NK cells is significantly lower in the blood of patients with high HBsAg levels and we validated this finding with flow cytometry. Gene expression profiles of this cluster matched with those of previously characterized NKG2C+ FcεRγ- NK cells, which are considered to be long-lived and presumably acquire memory-like properties and are therefore referred to as “adaptive” NK cells.^13^ Previous studies have described an expansion of NKG2C+ NK cells in various viral infections, and positive serology for CMV infection has been strongly associated with a higher proportion of NKG2C+ NK cells.^13^^,^^27^ In our patient cohort, a significant proportion of patients in both the high- and low-HBsAg groups had a positive CMV serology, indicating that CMV is unlikely to be the sole driver of our findings. To further validate this, cluster frequency and flow cytometry analysis in the CMV-positive subcohort demonstrated significantly higher frequencies of NKG2C+ NK cells in the HBsAg-low group, providing additional evidence supporting the association between HBsAg levels and NK cell frequency. Our results suggest that subset expansion of circulating *KLRC2*+ *NCAM1*^low^ “adaptive” NK cells is associated with HBsAg levels. This raises the question of why the frequency of this NK cell subpopulation is significantly lower in the blood of patients with high HBsAg levels. At the same time, together with the *KLRG1+* CD8 T cells, these cells display a more activated gene expression profile in the HBsAg-low group. Based on our observations, there is no indication that the equivalent of circulating *KLRC2*+ NK cells is present in the liver.

The role and presence of *KLRC2*+ *NCAM1*^*low*^ NK cells in HBV infection remain complex and not fully understood. Although their frequency is conserved in HBV infection,^28^ the association between *KLRC2*+ NK cells and HBsAg levels suggests an interaction between the two. Given the *ex vivo* nature of our study, prospective studies with sequential liver FNAs in patients with chronic HBV receiving drugs that modulate HBsAg levels are needed to investigate the impact of HBsAg on this population or vice versa. These studies could shed light on the effects of HBsAg on these cells and their role in the immune response against HBV. This includes exploring the possibility of antibody-dependent killing of HBsAg-presenting hepatocytes or HBsAg-mediated killing by *KLRC2*+ (NKG2C) NK cells in individuals with high HBsAg levels. It is also important to consider other potential factors, such as cell migration to lymphoid tissues other than the liver or reduced differentiation, that may contribute to the observed dynamics of this NK cell subset.

As expected and previously shown,^11^ serum HBsAg levels reflect HBsAg positivity in the liver where viral replication and protein synthesis occur. As in the blood, the effects of HBsAg in the liver were mainly restricted to NK-, B- and CD8 T-cell subsets. Importantly, we analyzed the immune cell landscape in both blood and liver samples and found no notable differences in the frequency of T-cell, B-cell, and antigen-presenting cell populations between patients with high and low levels of HBsAg. This indicates that HBsAg levels do not significantly influence the overall distribution of these immune cell subsets in the studied compartments. However, high HBsAg levels in the liver were associated with only minor gene expression changes, pointing towards mild immune activation. Gene expression profiles of *CXCR6+ CD69+* liver-resident CD8 T cells exhibited a differentiated effector phenotype that has been associated with tissue homing, enhanced cytotoxic characteristics, and repeated antigen stimulation.[24], [25], [26] Notably, in patients with chronic HBV and active liver damage, liver CXCR6+ CD8 T cells are enriched and exhibit a highly activated immune signature along with the expression of exhaustion-related markers.^29^ The expression of *CD69* in specific subgroups of immune cells suggests potential increased activation or tissue retention, which in turn may have implications for viral clearance and control; however, these findings need to be confirmed in longitudinal prospective studies. In our study, in which NUC-treated patients were studied, we found no evidence supporting a role for exhausted T-cell phenotypes, as observed in liver scRNAseq studies focusing on active hepatitis.^29^^,^^30^ It is important to note that our data does not demonstrate induction of gene expression patterns associated with immune inhibition in the HBsAg-high group. Thus, we do not suggest a terminally dysfunctional or exhausted differentiation of CD8 T cells as observed in chronic viral infections.^31^ In addition, memory B cells showed signs of activation in both the blood and liver of patients with high HBsAg levels. It is important to note that our analysis focused on the impact of high and low HBsAg levels on immune cell subsets. To specifically investigate the impact of HBsAg levels, we did not include healthy control samples in our study. However, for future studies aiming to understand the broader effects of HBsAg and HBV-related particles, it may be useful to compare publicly available datasets with our findings.

In order to get a better understanding of the effects of HBsAg, our cohort included patients receiving long-term NUC treatment, with undetectable HBV DNA levels, and no significant fibrosis. Despite successful suppression of HBV by NUC treatment, signs of immune activation were observed in patients with relatively high HBsAg levels. This further brings into question whether these signs of low-grade inflammation in the liver have any consequences for patient health or long-term outcome. Furthermore, it remains unknown whether these signs of HBsAg-associated low-grade liver inflammation could also be observed in inactive carrier patients who are able to control viral replication naturally.

Our study provides valuable insight into the impact of HBsAg on immune cell subsets, especially in the context of NUC-treated HBV. While studying patients with active hepatitis offers insights into the immune response during inflammation, NUC-treated patients are highly relevant for clinical trials. Although we acknowledge the need for caution in interpreting causality, our study sheds light on the potential influence of prolonged HBsAg exposure on HBV-specific immune responses mediated by CD8+ T cells or memory B cells during NUC-treated chronic HBV infection. Thus, our findings hold significant relevance for current and future clinical studies aimed at developing novel treatment strategies to suppress HBsAg production and enhance the antiviral response against HBV.

Our findings raise questions about the effectiveness of strategies targeting HBsAg production, as our analysis indicates that antigen-presenting cells, such as dendritic cells and Kupffer cells, do not appear to be significantly affected by HBsAg. Furthermore, we demonstrate that relatively high levels of HBsAg in the liver are associated with low-grade inflammation rather than immune inhibition or exhaustion-related gene profiles. Taken together, these findings challenge the previously hypothesized immunomodulatory effects of HBsAg on immune responses against HBV and underscore the need for further investigation to fully understand the complex interactions between HBsAg and the immune system, particularly in the context of novel therapeutic interventions aimed at HBV suppression.

## Financial support

10.13039/501100015383The Foundation for Liver and Gastrointestinal Research (SLO) sponsored the study. The funding source did not influence the study design, data collection, analysis and interpretation of the data, writing of the report, or the decision to submit for publication.

## Authors’ contributions

B.J.B.B. and A.B. contributed to the design of the research. B.J.B.B. collected clinical data and patient samples. G.W.O. and Z.M.A.G. carried out the experiments and procedures, R.M.H. and E.M.J.B. performed the sequencing. R.J.K., H.J.G.W. and A.B. were involved in planning and supervised the work. Z.O. was responsible for conducting the bioinformatic analysis. Z.O. wrote the manuscript. B.J.B.B. and A.B. contributed to the analysis of the results and co-wrote the manuscript. All authors discussed the results, provided critical feedback and contributed to the final manuscript.

## Data availability statement

Data is publically available and has been deposited in GEO (https://www.ncbi.nlm.nih.gov/geo/) with accession number: GSE247322.

## Conflict of interest

A.B. received grants not related to this project from Gilead Sciences, Fujirebio, GlaxoSmithKline, and Janssen Pharma. R.J.K. received grants from GlaxoSmithKline, Janssen-Cilag, and Echosens (not related to this project), and received consulting fees, payments or honoraria for lectures, presentations, or other educational events from AbbVie, Echosens, and Gilead Sciences. All remaining authors declare that they have no conflicts of interest.

Please refer to the accompanying ICMJE disclosure forms for further details.
