# Supplementary material for: Association of HBsAg levels with differential gene expression in NK, CD8 T, and memory B cells in treated patients with chronic HBV
Source: JHEP Rep. 2023 Dec 3;6(2):100980. doi: 10.1016/j.jhepr.2023.100980 (PMC10835465; doi:10.1016/j.jhepr.2023.100980)
Supplement: Multimedia component 1 [file mmc1.pdf]

Association of HBsAg levels with differential gene expression in NK, CD8 T, and memory B cells in treated patients with chronic HBV

Boris J.B. Beudeker, **Zgjim Osmani**, Gertine W. van Oord, Zwier M.A. Groothuismink, Robert J. de Knegt, Remco M. Hoogenboezem, Eric M.J. Bindels, Harmen J.G. van de Werken, Andre Boonstra

Table of contents

Supplementary figures 2

Supplementary tables……………………………………………………….10

Supplementary figures

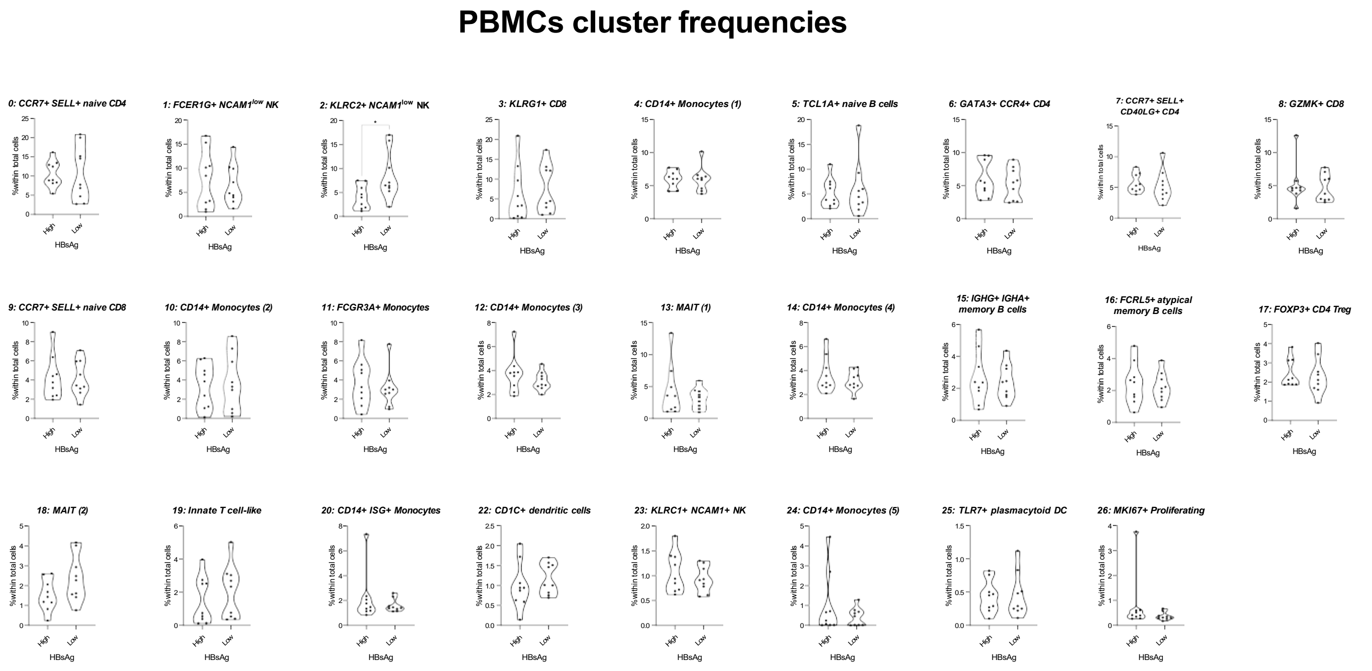

Fig. S1. Overview of PBMCs cluster frequencies comparing HBsAg high vs. low, relative frequencies are shown as %within total cells.

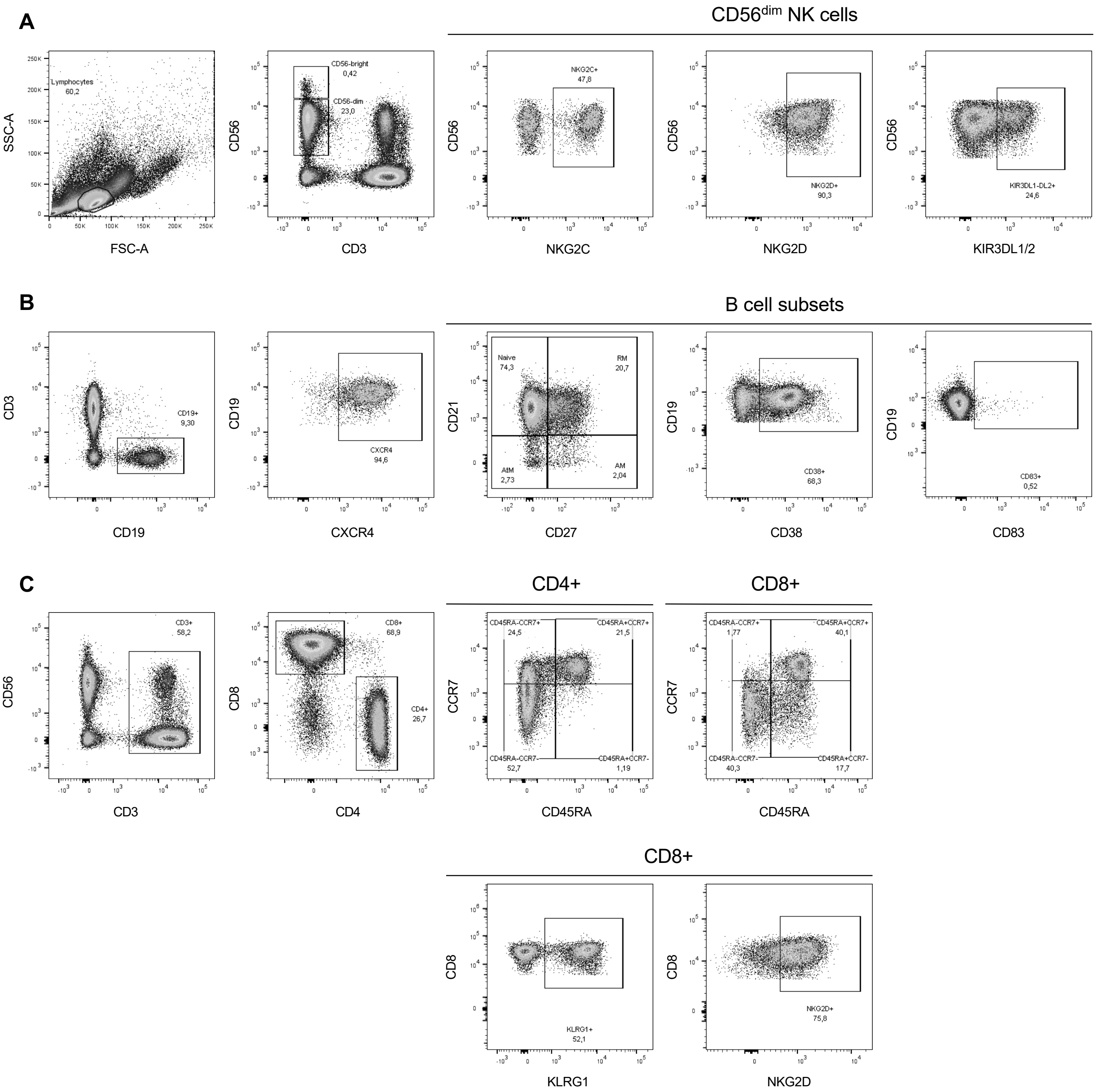

Fig. S2. Representative FACS plots and gating strategy showing the flow cytometry analysis of viable lymphocytes and gating of CD56+ NK cell (A), CD19+ B cell (B), and CD4/CD8+ T cell subsets (C). Cells were stained with surface markers to asses the distribution, maturation and activation status of each subset.

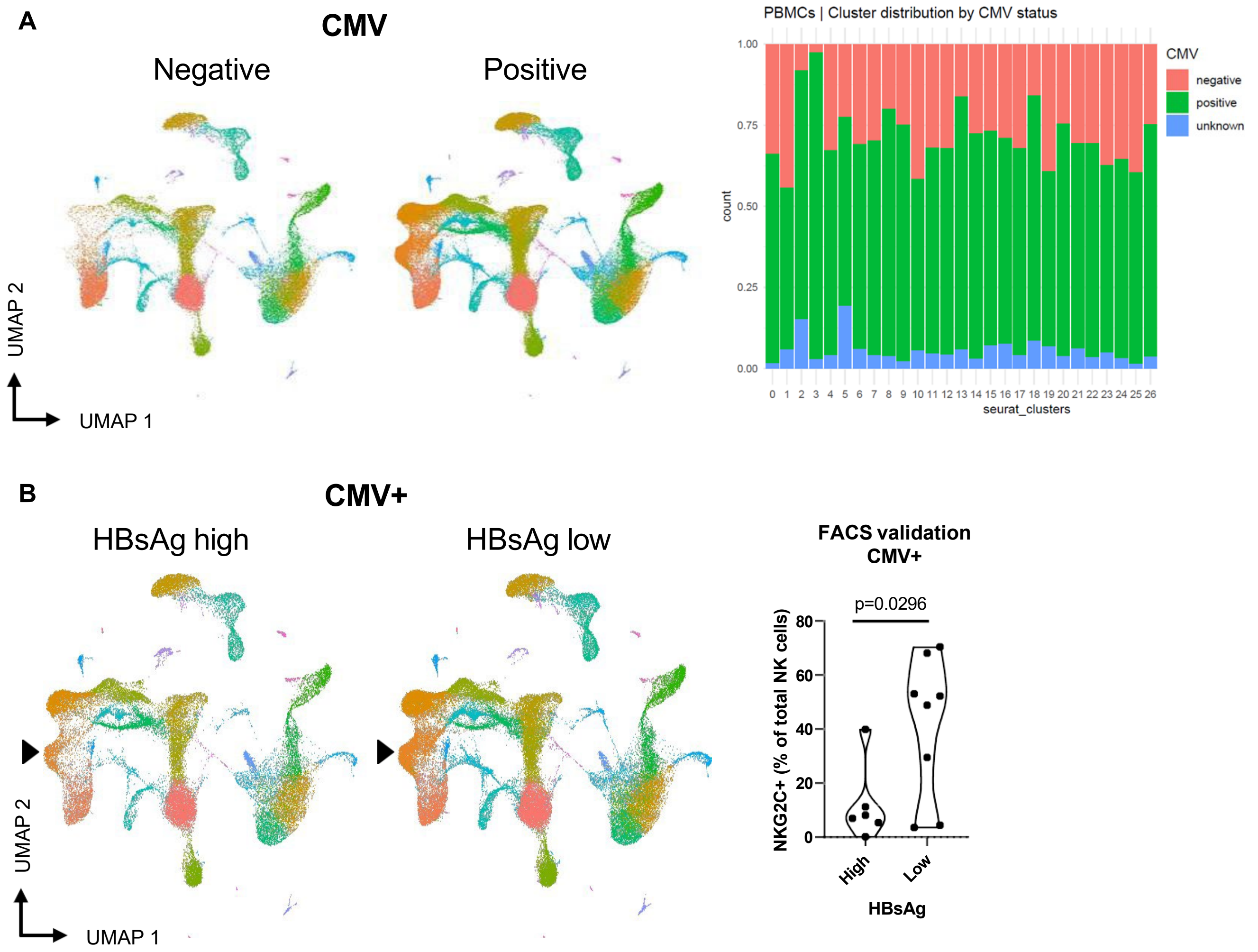

Fig. S3. UMAP split by CMV status and bar plot showing the distribution of all clusters identified in peripheral blood (A). Extended subgroup analysis focused specifically on the frequency of NKG2C+ NK cells in the blood of CMV+ chronic HBV patients (B). There is a significant association between the frequency of NKG2C+ NK cells (black arrow) and HBsAg status, validated by flow cytometry. We observed a median frequency of 7.5% (IQR: 4.6) in the HBsAg high group and 50.5% (IQR: 33.5) among the HBsAg low group (p=0.0296).

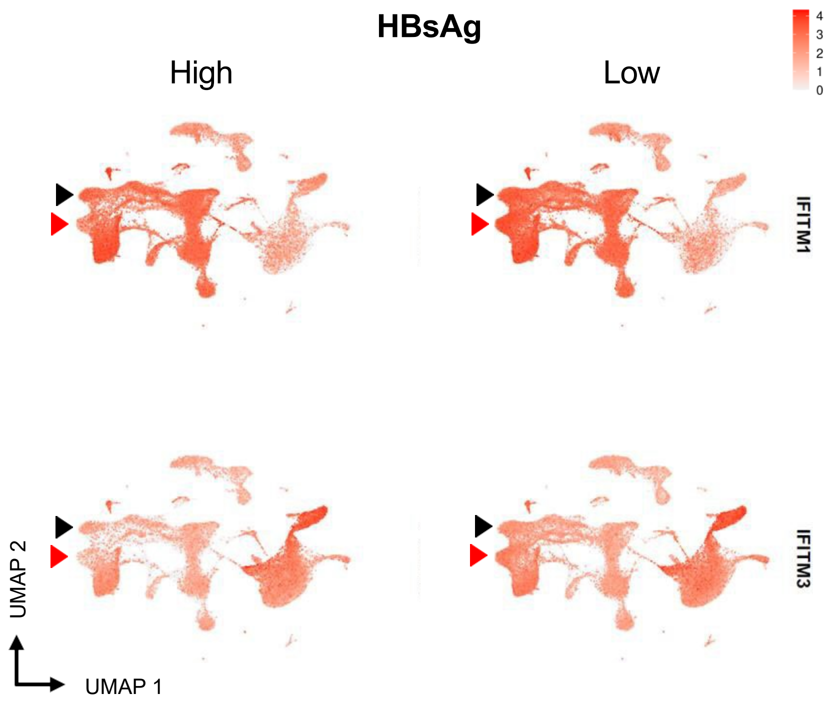

Fig. S4. Feature plot showing gene expression levels of *IFITM1* and *IFITM3* in peripheral blood clusters, split by HBsAg level. Each dot represents a single cell, and gene expression values are indicated by a color scale. As shown in the feature plots, it is evident that in HBsAg low samples, the *KLRC2*+ NK cell (red arrow) and *KLRG1*+ CD8 T cell cluster (black arrow) exhibit higher expression levels of *IFITM1* and *IFITM3*. These populations are found on the left side of the UMAP plots.

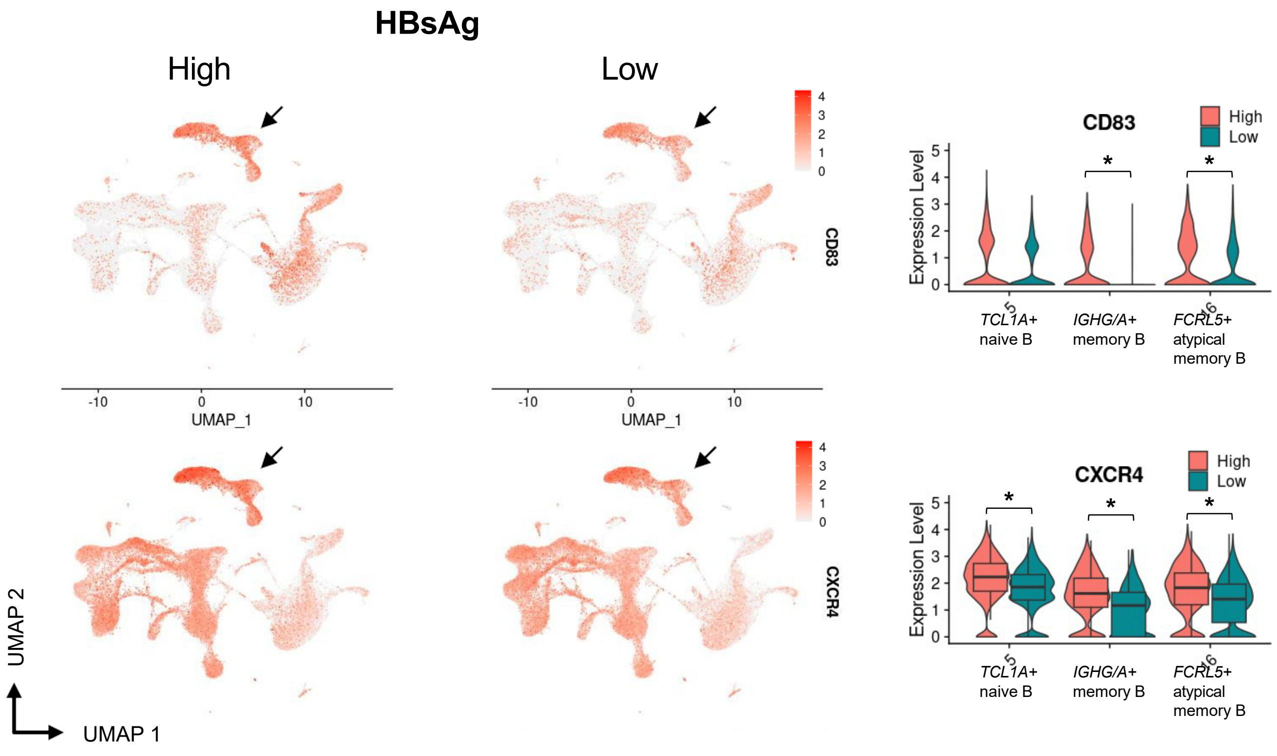

Fig. S5. Feature plot split by HBsAg level showing gene expression levels of *CD83* and *CXCR4* in all clusters identified in peripheral blood. Each dot represents a single cell, gene expression values are represented by a color scale. The black arrow points towards the B cell clusters. Gene expression levels are shown in violin plots on the right side of the figure for the *TCL1A*+ naive B cell, *IGHG/A*+ memory B cell, and *FCRL5*+ atypical memory B cell cluster.

FNAs cluster frequencies

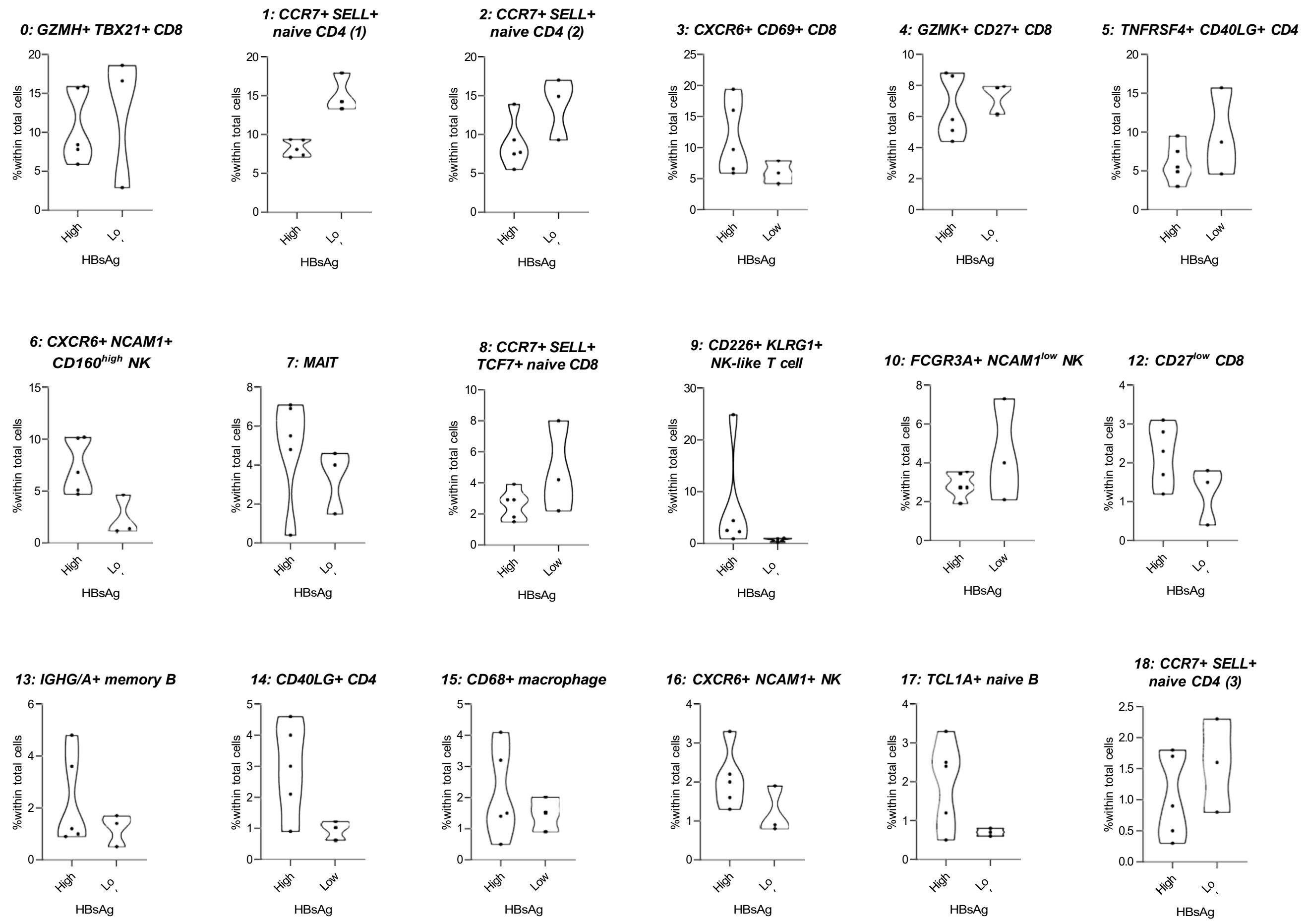

Fig. S6. Overview of FNAs cluster frequencies comparing HBsAg high vs. low, relative frequencies are shown as %within total cells.

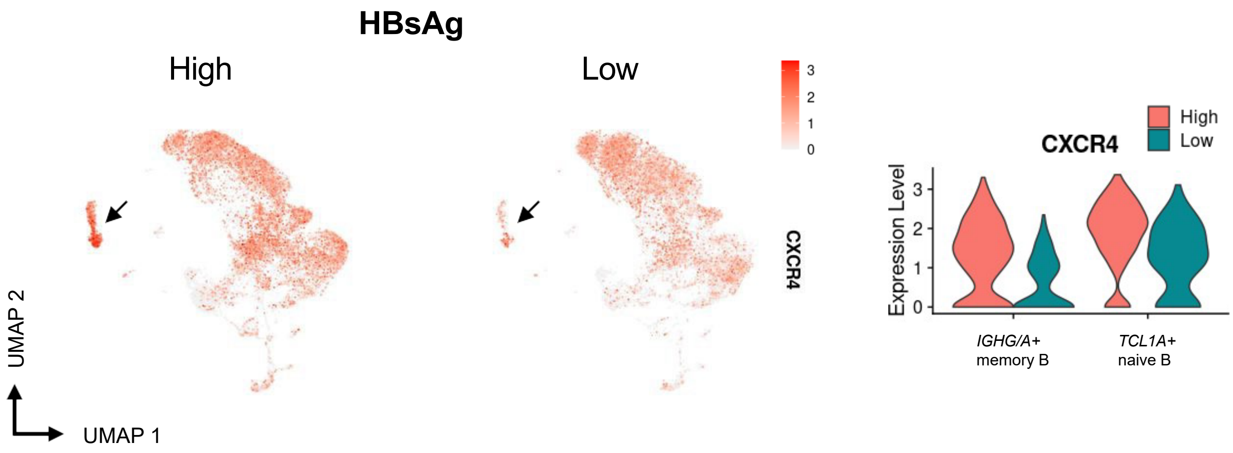

Fig. S7. Feature plot split by HBsAg level showing gene expression level of *CXCR4* in all clusters identified in liver FNAs. Each dot represents a single cell, gene expression values are represented by a color scale. The black arrow points towards the B cell clusters. Gene expression levels are shown in violin plots on the right side of the figure for the *TCL1A+* naive B cell and *IGHG/A+* memory B cell cluster.

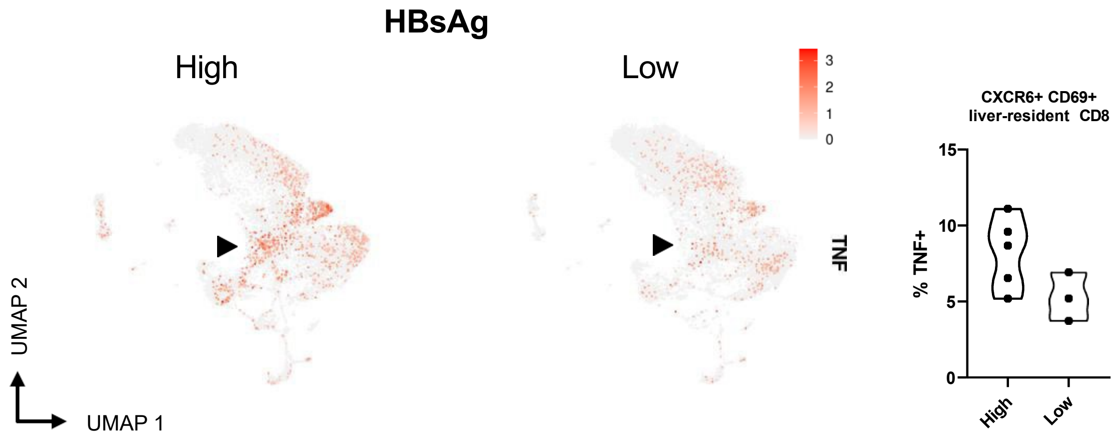

Fig. S8. Feature plot split by HBsAg level showing gene expression level of *TNF* in all clusters identified in liver FNAs. Each dot represents a single cell, gene expression values are represented by a color scale. The black arrow points towards the *CXCR6+ CD69+* liver-resident CD8 T cell cluster. On the right side of the figure, the percentage of cells that express *TNF* are shown in a violin plot.

Supplementary tables

Table S1. Quality control and filtering of PBMCs scRNAseq data.

		**Raw data**			**Count data**	**Filtered data**	**ΔCounts**
**Sample**	**HBsAg Group**	Estimated number of cells	Mean reads/cell	Median genes/cell	Number of cells with >200 features	Number of cells with <20% mito, >600 & <4000 features	% loss
**pbmc1**	High	7,019	27,567	1,480	6,466	5,831	9.8
**pbmc4**	High	6,289	30,117	1,674	6,254	5,750	8.1
**pbmc5**	High	6,787	19,918	1,453	6,768	6,217	8.1
**pbmc8**	High	9,209	20,502	1,372	9,147	7,677	16.1
**pbmc10**	High	6,308	58,760	2,199	6,266	5,842	6.8
**pbmc12**	High	9,913	11,917	1,501	9,905	9,623	2.8
**pbmc14**	High	14,627	8,748	1,294	14,615	14,292	2.2
**pbmc17**	High	9,157	14,617	1,731	9,131	8,840	3.2
**pbmc18**	High	12,323	9,298	1,288	12,320	11,893	3.5
**pbmc3**	Low	6,882	31,246	1,616	6,815	6,285	7.8
**pbmc6**	Low	9,051	25,554	1,655	8,822	8,042	8.8
**pbmc7**	Low	13,805	49,376	1,858	13,749	12,956	5.8
**pbmc9**	Low	7,770	34,037	1,484	7,638	7,367	3.5
**pbmc13**	Low	12,458	13,641	1,586	12,446	11,904	4.4
**pbmc15**	Low	9,994	13,853	1,654	9,988	9,760	2.3
**pbmc16**	Low	10,484	10,906	1,477	10,462	10,082	3.6
**pbmc19**	Low	13,189	8,332	1,229	13,187	12,886	2.3
**pbmc20**	Low	11,447	12,615	1,632	11,441	11,104	2.9

Table S2. PBMCs differential gene expression analysis for each cluster.

Significant differential expressed genes (DEGs) were identified for each cluster in the PBMCs dataset. Results per cluster can be found within separate sheets in a large excel file. The average log2FC and (adjusted) p values are shown in seperate columns.

Table S3. PBMCs cell frequency of each cluster.

		**Count**			**Percentage**	
**Cluster**	**Cell type**	Total	HBsAg High	HBsAg Low	HBsAg High	HBsAg Low
**0**	*CCR7+ SELL+* naive CD4	17,568	8,209	9,359	10.81	10.35
**1**	*FCER1G+ NCAM1*^{low} NK	11,905	6,122	5,783	8.06	6.40
**2**	*KLRC2+ NCAM1*^{low} NK	10,215	2,604	7,611	3.43	8.42
**3**	*KLRG1+* CD8	10,266	3,816	6,450	5.02	7.14
**4**	*CD14+* Monocytes (1)	10,161	4,569	5,592	6.01	6.19
**5**	*TCL1A+* naive B cells	9,591	4,327	5,264	5.70	5.82
**6**	*GATA3+ CCR4+* CD4	9,481	4,856	4,625	6.39	5.12
**7**	*CCR7+ SELL+ CD40LG+* CD4	9,169	4,442	4,727	5.85	5.23
**8**	*GZMK+* CD8	7,774	3,625	4,149	4.77	4.59
**9**	*CCR7+ SELL+* naive CD8	6,688	3,107	3,581	4.09	3.96
**10**	*CD14+* Monocytes (2)	6,815	3,052	3,763	4.02	4.16
**11**	*FCGR3A+* Monocytes	5,822	2,891	2,931	3.81	3.24
**12**	*CD14+* Monocytes (3)	5,754	2,860	2,894	3.76	3.20
**13**	MAIT (1)	5,603	2,775	2,828	3.65	3.13
**14**	*CD14+* Monocytes (4)	5,465	2,588	2,877	3.41	3.18
**15**	*IGHG+ IGHA+* memory B cells	4,411	2,124	2,287	2.80	2.53
**16**	*FCRL5+* atypical memory B cells	4,004	1,974	2,030	2.60	2.25
**17**	*FOXP3+* CD4 Treg	3,985	1,931	2,054	2.54	2.27
**18**	MAIT (2)	3,349	1,175	2,174	1.55	2.41
**19**	Innate T cell-like	3,371	1,392	1,979	1.83	2.19
**20**	*CD14+ ISG+* Monocytes	2,966	1,495	1,471	1.97	1.63
**22**	*CD1C+* dendritic cells	1,911	808	1,103	1.06	1.22
**23**	*KLRC1+* NCAM1+ NK	1,711	836	875	1.10	0.97
**24**	*CD14+* Monocytes (5)	1,408	973	435	1.28	0.48
**25**	*TLR7+* plasmacytoid DC	821	364	457	0.48	0.51
**26**	*MKI67+* Proliferating	850	514	336	0.68	0.37

Sample distribution among the obtained PBMCs clusters.

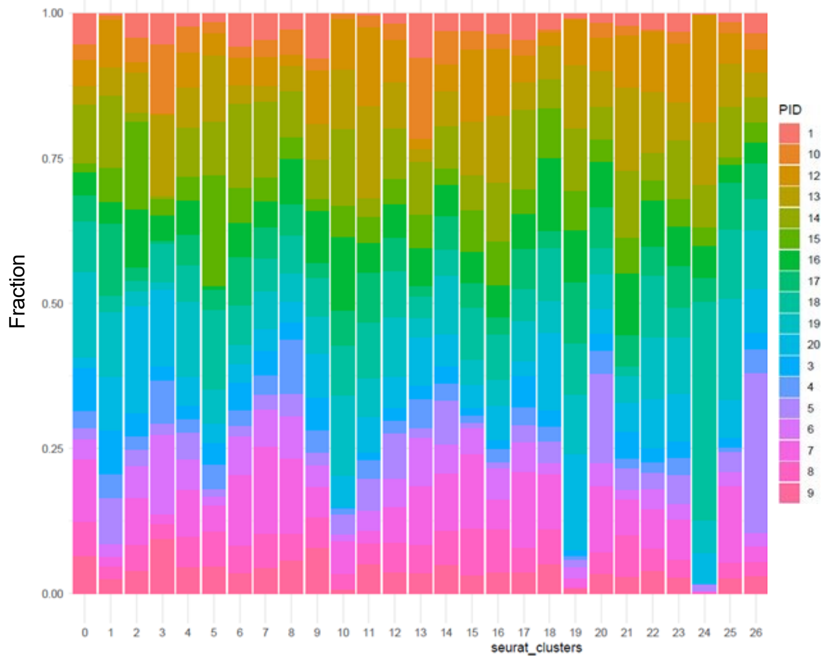

Table S4. Overview of FACS validation results. Flow cytometric analyses were performed on B-, NK-, CD4-, and CD8 T cell subsets in blood of patients with high vs. low HBsAg levels to asses the distribution, maturation and activation status of each subset. Results are summarized as median (IQR) percentages.

Table S5. PBMCs differential gene expression analysis comparing clusters between patients with high HBsAg vs. low HBsAg levels.

Significant differential expressed genes (DEGs) were identified for each circulating cell cluster comparing HBsAg high vs. low. Results per cluster can be found within separate sheets in a large excel file. The average log2FC and (adjusted) p values are shown in seperate columns. Only significant results (p.adj <0.05) with an absolute fold change of 1.5 are shown.

Table S6. PBMCs gene set enrichment analysis for *KLRC2*+ *NCAM1*^{low} NK cells and *KLRG1*+ CD8 T cells.

Significant gene sets (p.adj <0.05) were identified for *KLRC2*+ *NCAM1*^{low} NK cells and *KLRG1*+ CD8 T cells comparing gene expression profiles of these clusters between HBsAg high vs. low. Results per cluster can be found within separate sheets in an excel file. The normalized enrichment score (NES) and (adjusted) p values are shown in separate columns.

Table S7. Quality control and filtering of FNAs scRNAseq data.

		Raw data			Count data	Filtered data	ΔCounts
**Sample**	**HBsAg Group**	Estimated number of cells	Mean reads/cell	Median genes/cell	Number of cells with > 200 features	Number of cells with <20% mito, > 600 and < 4000 features	% loss
**fnap1**	High	2,741	20,992	872	2,349	1,395	40.61
**fnap4**	High	5,842	20,571	1,316	5,748	3,498	39.14
**fnap5**	High	5,157	20,992	1,269	5,135	3,636	29.19
**fnap8**	High	12,846	32,975	1,343	12,601	7,172	43.08
**fnap10**	High	6,224	53,564	1,778	6,190	3,808	38.48
**fnap6**	Low	9,642	20,590	1,355	9,317	5,721	38.60
**fnap7**	Low	8,799	18,758	810	8,787	2,600	70.41
**fnap9**	Low	10,196	25,830	1,506	9,960	7,683	22.86

Table S8. FNAs differential gene expression analysis for each cluster.

Significant differential expressed genes (DEGs) were identified for each cluster in the FNAs dataset. Results per cluster can be found within separate sheets in a large excel file. The average log2FC and (adjusted) p values are shown in seperate columns.

Table S9. Patient characteristics, selection of fine-needle aspirates.

	Unit	High	Low	P value
Patients	N	5	3	
Age (years)	Median (IQR)	43 (23)	52 (9)	0.393
Sex (male)	N, %	5 (100%)	3 (100%)	
HBeAg negative	N, %	5 (100%)	3 (100%)	
Log HBV DNA (IU/mL)	Median (IQR)	Undetectable (<20)	Undetectable (<20)	
ALT (U/L)	Median (IQR)	21 (34)	24 (14)	0.764
HBsAg (IU/mL)	Median (IQR)	4600 (3985)	70 (31)	0.036
Treatment regime	N, %			0.850
TDF		3 (60%)	2 (66%)	
ETV		2 (40%)	1 (33%)	
Treatment duration (years)	Median (IQR)	4 (2)	9 (2)	0.250
F0/F1 fibrosis stage	N, %	5 (100%)	3 (100%)	
Anti-CMV IgG	+ / - / ND	5 / 0 / 0	3 / 0 / 0	
Anti-HBe	+ / - / ND	5 / 0 / 0	3 / 0 / 0	
Anti-HBs	+ / - / ND	0 / 5 / 0	0 / 3 / 0	

Abbreviations: IQR, interquartile range; TDF, tenofovir disoproxil fumarate; ETV, entecavir; CMV, cytomegalovirus; ND: not determined.

Table S10. FNAs cell frequency of each cluster.

Cluster	Cell type	Count			Percentage	
		Total	HBsAg High	HBsAg Low	HBsAg High	HBsAg Low
**0**	*GZMH+ TBX21+* CD8	4,395	1,980	2,415	10.15	15.09
**1**	*CCR7+ SELL+* naive CD4 (1)	4,172	1,639	2,533	8.40	15.83
**2**	*CCR7+ SELL+* naive CD4 (2)	3,626	1,551	2,075	7.95	12.97
**3**	*CXCR6+ CD69+* CD8	3,463	2,427	1,036	12.44	6.47
**4**	*GZMK+ CD27+* CD8	2,437	1,226	1,211	6.28	7.57
**5**	*TNFRSF4+ CD40LG+* CD4	2,337	1,079	1,258	5.53	7.86
**6**	*CXCR6+ NCAM1+ CD160*^{high} NK	1,820	1,357	463	6.96	2.89
**7**	MAIT	1,574	1,034	540	5.30	3.37
**8**	*CCR7+ SELL+ TCF7+* naive CD8	1,447	536	911	2.75	5.69
**9**	*CD226+ KLRG1+* NK-like T cell	1,433	1,296	137	6.64	0.86
**10**	*FCGR3A+ NCAM1*^{low} NK	1,156	536	620	2.75	3.87
**12**	*CD27*^{low} CD8	715	476	239	2.44	1.49
**13**	*IGHG/A+* memory B	679	508	171	2.60	1.07
**14**	*CD40LG+* CD4	678	537	141	2.75	0.88
**15**	*CD68+* macrophage	667	464	203	2.38	1.27
**16**	*CXCR6+ NCAM1+* NK	637	420	217	2.15	1.36
**17**	*TCL1A+* naive B	532	415	117	2.13	0.73
**18**	*CCR7+ SELL+* naive CD4 (3)	509	232	277	1.19	1.73

Sample distribution among the obtained FNAs clusters.

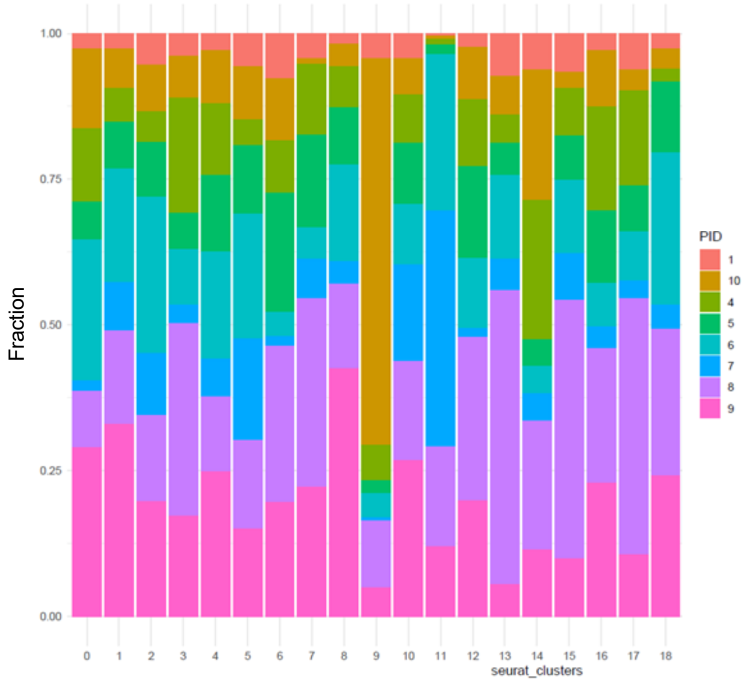

Table S11. FNAs differential gene expression analysis comparing clusters between patients with high HBsAg vs. low HBsAg levels.

Significant differential expressed genes (DEGs) were identified for each intrahepatic cell cluster comparing HBsAg high vs. low. Results per cluster can be found within separate sheets in a large excel file. The average log2FC and (adjusted) p values are shown in seperate columns. Only significant results (p.adj <0.05) with an absolute fold change of 1.5 are shown.

Table S12. FNAs gene set enrichment analysis for liver-resident *CXCR6*+ *CD69*+ CD8 T cells.

Significant gene sets (p.adj <0.05) were identified for *CXCR6*+ *CD69*+ liver-resident CD8 T cells comparing gene expression profiles of these clusters between HBsAg high vs. low. Results can be found in an excel file. The normalized enrichment score (NES) and (adjusted) p values are shown in separate columns.
