# Supplementary material for: Association of HBsAg levels with differential gene expression in NK, CD8 T, and memory B cells in treated patients with chronic HBV
Source: JHEP Rep. 2023 Dec 3;6(2):100980. doi: 10.1016/j.jhepr.2023.100980 (PMC10835465; doi:10.1016/j.jhepr.2023.100980)
Supplement: Multimedia component 2 — : [file mmc2.docx]

**Journal of Hepatology**

**CTAT methods**

Tables for a “Complete, Transparent, Accurate and Timely account” (CTAT) are now mandatory for all revised submissions. The aim is to enhance the reproducibility of methods.

- Only include the parts relevant to your study
- Refer to the CTAT in the main text as ‘Supplementary CTAT Table’
- Do not add subheadings
- Add as many rows as needed to include all information
- Only include one item per row

**If the CTAT form is not relevant to your study, please outline the reasons why:**

|  |
| --- |

- 1. **Antibodies**

| **Name** | **Supplier** | **Cat no.** | **Clone no.** |
| --- | --- | --- | --- |
| CD19 APC-Fire750 | BioLegend | 302258 | HIB19 |
| CD3 BV570 | BioLegend | 300436 | UCHT1 |
| CD21 PE-Dazzle594 | BioLegend | 354921 | Bu32 |
| CD27 FITC | BD Biosciences | 340424 | L128 |
| CD38 PerCP-eFluor710 | eBiosciences | 46-0388-42 | HB7 |
| CD83 PE | BioLegend | 305322 | HB15e |
| CD19 eF450 | eBiosciences | 48-0199-42 | HIB19 |
| CXCR4 PE | BioLegend | 306506 | 12G5 |
| CD3 eF450 | eBiosciences | 48-0037 | OKT3 |
| CD3 PE | BioLegend | 300408 | UCHT1 |
| CD8 eF450 | eBiosciences | 48-0088-42 | RPA-T8 |
| CD56 APC | eBiosciences | 17-0567 | CMSSB |
| NKG2C Alexa Fluor488 | R&D Systems | FAB138G | 134591 |
| NKG2D PerCP-Cy5.5 | BD Biosciences | 562364 | ID11 |
| KIR3DL1/2 biotin | Miltenyi Biotec | 130-095-201 | 5133 |
| Streptavidin PE | BioLegend | 405203 | n/a |
| CD3 Amcyan | BD Biosciences | 339186 | SK7 |
| CD56 APC-Vio770 | Miltenyi Biotec | 130-114-548 | REA196 |
| CD4 PE-eFluor610 | eBiosciences | 61-0049-42 | RPA-T4 |
| CD8 Super Bright 600 | eBiosciences | 63-0088-42 | RPA-T8 |
| CD45RA FITC | eBiosciences | 11-0458-42 | JI100 |
| CCR7 BV750 | BioLegend | 353254 | G043H7 |
| KLRG1 PE | BioLegend | 367712 | SA231A2 |

- 1. **Cell lines**

| **Name** | **Citation** | **Supplier** | **Cat no.** | **Passage no.** | **Authentication test method** |
| --- | --- | --- | --- | --- | --- |
|  |  |  |  |  |  |

- 1. **Organisms**

| **Name** | **Citation** | **Supplier** | **Strain** | **Sex** | **Age** | **Overall n number** |
| --- | --- | --- | --- | --- | --- | --- |
|  |  |  |  |  |  |  |

- 1. **Sequence based reagents**

| **Name** | **Sequence** | **Supplier** |
| --- | --- | --- |
| Single Cell 3’ v3 Reagent Kit | n/a | 10x Genomics |
| Single Cell 5’ v2 Reagent Kit | n/a | 10x Genomics |

- 1. **Biological samples**

| **Description** | **Source** | **Identifier** |
| --- | --- | --- |
| Human immune cells | Peripheral blood | n/a |
| Human intrahepatic immune cells | Liver fine-needle aspirates | n/a |

- 1. **Deposited data**

| **Name of repository** | **Identifier** | **Link** |
| --- | --- | --- |
| T.B.A. | T.B.A. | T.B.A. |

- 1. **Software**

| **Software name** | **Manufacturer** | **Version** |
| --- | --- | --- |
| R | R Core Team | 4.2.2 |

- 1. **Other (e.g. drugs, proteins, vectors etc.)**

|  |  |  |
| --- | --- | --- |
|  |  |  |

- 1. **Please provide the details of the corresponding methods author for the manuscript:**

| Prof. dr. Andre Boonstra E-mail: [p.a.boonstra@erasmusmc.nl](mailto:p.a.boonstra@erasmusmc.nl) Address: Wytemaweg 80, 3015 CN Rotterdam, post office 2040, 3000 CA Rotterdam, The Netherlands. |
| --- |

**2.0 Please confirm for randomised controlled trials all versions of the clinical protocol are included in the submission. These will be published online as supplementary information.**

|  |
| --- |
