# Supplementary material for: Association of HBsAg levels with differential gene expression in NK, CD8 T, and memory B cells in treated patients with chronic HBV
Source: JHEP Rep. 2023 Dec 3;6(2):100980. doi: 10.1016/j.jhepr.2023.100980 (PMC10835465; doi:10.1016/j.jhepr.2023.100980)
Supplement: Multimedia component 4 [file mmc4.pdf]

Supplemental Data 1

Clustering of PBMCs

We aimed to identify specific immune cell clusters in blood of patients with chronic HBV that could be affected by HBsAg. First, we identified all immune subsets in peripheral blood (Suppl. Data 1A). After quality control and filtering, a median of 9,232 high-quality cells were sequenced per patient sample (Suppl. Table 1). Clustering of 166,351 PBMCs identified 36 distinct cell clusters in peripheral blood. Platelets and clusters with less than 500 cells were excluded from downstream analysis, the remaining 27 clusters were found in all patient samples. Clusters were annotated according to differentially expressed genes (Suppl. Data 1B-C, Suppl. Table 2). We identified a total of four CD4 T cell clusters, including *CCR7*+ *SELL*+ naive CD4, *CCR7*+ *SELL*+ *CD40LG*+ CD4, *GATA3*+ *CCR4*+ CD4, and a *FOXP3*+ CD4 T regulatory cell cluster. In addition, we found three CD8 T cell clusters in peripheral blood: *CCR7*+ *SELL*+ naive CD8, *GZMK*+ CD8 and a *KLRG1*+ CD8 T cell cluster. Monocytes were divided into seven distinct clusters, including five *CD14*+ clusters (monocyte 1-5) and two unique clusters of *FCGR3A*+ monocytes and *CD14*+ *ISG*+ monocytes characterized by the upregulation of multiple interferon-stimulated genes (ISG). Furthermore, three NK cell clusters were characterized: *FCER1G*+ *NCAM1*^{low} NK, *KLRC2*+ *NCAM1*^{low} NK, and a *KLRC1*+ *NCAM1*+ NK cell cluster, and three B cell clusters: *TCL1A*+ naive, *IGHG*+ *IGHA*+ memory, and a *FCRL5*+ atypical memory B cell cluster. Less frequent cell clusters, such as an innate T cell-like cluster, *CD1C*+ dendritic cell cluster, *TLR7*+ plasmacytoid dendritic cell cluster, *MKI67*+ proliferating cell cluster, and two MAIT cell clusters were also identified. Clustering of PBMCs from chronic HBV patients identified distinct immune cell clusters, including multiple CD4-, CD8-, NK-, B cell, and monocyte subsets, providing a comprehensive picture of innate and adaptive immune cell populations in blood.

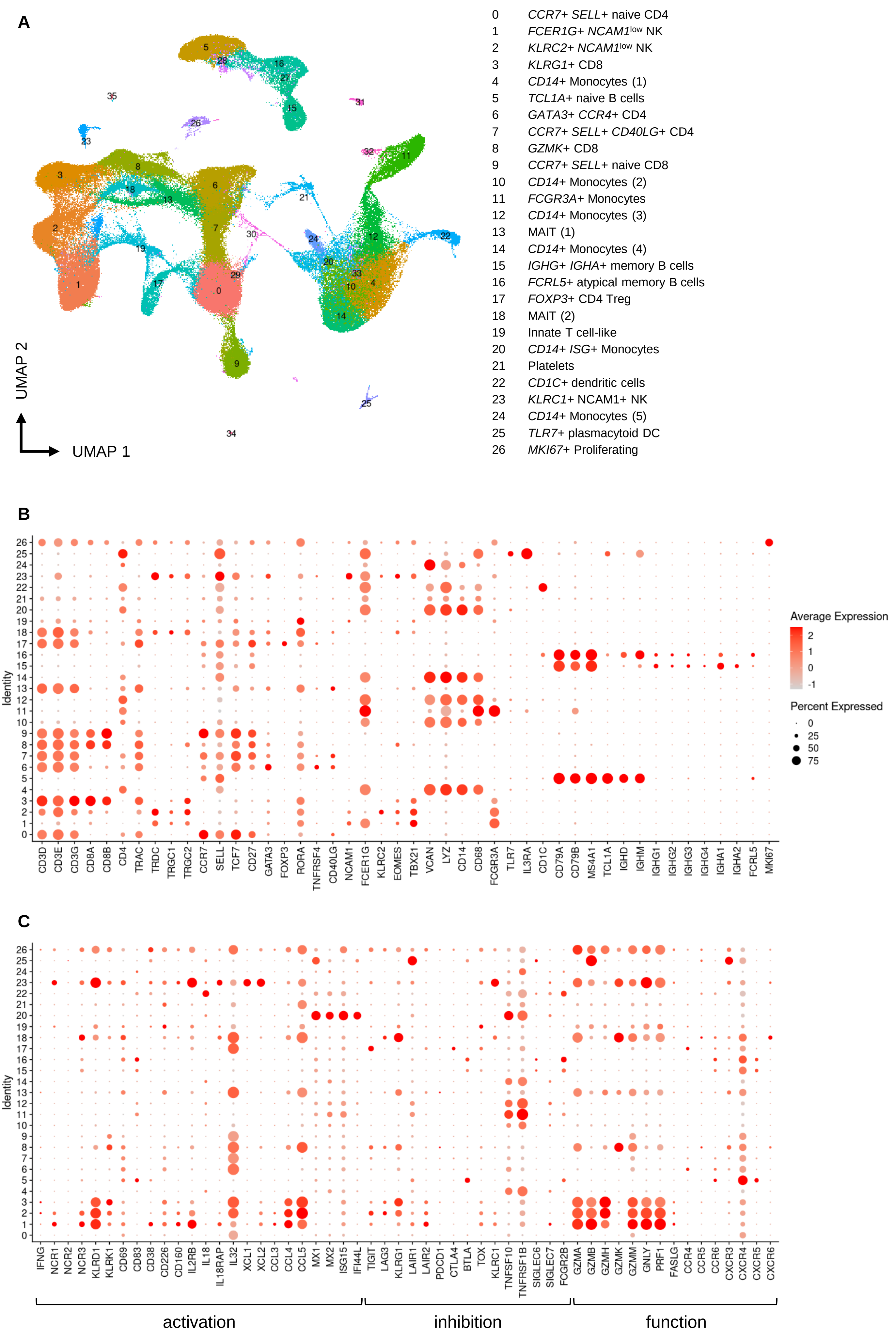

Supplemental Data 1. Clustering of PBMCs (n=166,351). (A) UMAP clustering of 36 cell clusters and annotation of 27 clusters with >500 cells. (B) Differential expressed genes and cell-type specific markers for each cluster. (C) Gene expression markers associated with immune activation, inhibition and functional markers. Color intensity and size of dots represents gene expression level and the percentage of cells expressing the gene of interest, respectively.

Signature genes of clusters in PBMCs

No.	Cluster	Signature genes							
0	CCR7+ SELL+ naive CD4	CD3D	CD3E	CD3G	CD4	CCR7	SELL		
1	FCER1G+ NCAM1low NK	GNLY	NKG7	PRF1	GZMB	GZMA	IL2RB	FCER1G	
2	KLRC2+ NCAM1low NK	GNLY	NKG7	PRF1	GZMB	GZMA	KLRC2	FCGR3A	
3	KLRG1+ CD8	CD3D	CD3E	CD3G	CD8A	CD8B	KLRG1		
4	CD14+ Monocytes (1)	LYZ	VCAN	S100A8	S100A9	CD14	CD68		
5	TCL1A+ naive B cells	TCL1A	MS4A1	CD79A	CD79B	IGHM	IGHD	FCER2	
6	GATA3+ CCR4+ CD4	CD3D	CD3E	CD3G	CD4	GATA3	CCR4		
7	CCR7+ SELL+ CD40LG+ CD4	CD3D	CD3E	CD3G	CD4	CCR7	SELL	CD40LG	
8	GZMK+ CD8	CD3D	CD3E	CD3G	CD8A	CD8B	GZMK		
9	CCR7+ SELL+ naive CD8	CD3D	CD3E	CD3G	CD8A	CD8B	CCR7	SELL	
10	CD14+ Monocytes (2)	LYZ	VCAN	S100A8	S100A9	CD14	CD68		
11	FCGR3A+ Monocytes	FCGR3A	CD68	LYZ	FCER1G				
12	CD14+ Monocytes (3)	LYZ	VCAN	S100A8	S100A9	CD14	CD68		
13	MAIT (1)	CD3D	CD3E	CD3G	IL7R	RORA	TRAC	KLRB1	
14	CD14+ Monocytes (4)	LYZ	VCAN	S100A8	S100A9	CD14	CD68		
15	IGHG+ IGHA+ memory B cells	MS4A1	CD79A	CD79B	AIM2	IGHG1-4	IGHA1-2		
16	FCRL5+ atypical memory B cells	MS4A1	CD79A	CD79B	FCRL5	IGHD	IGHM	IGHG1-3	IGHA1
17	FOXP3+ CD4 Treg	CD3D	CD3E	CD3G	CD4	FOXP3			
18	MAIT (2)	CD3D	CD3E	CD3G	IL7R	RORA	KLRB1		
19	Innate T cell-like	CD247	RORA	CD3E					
20	CD14+ ISG+ Monocytes	LYZ	VCAN	S100A8	S100A9	CD14	MX1	ISG15	IFI30
21	Platelets	PPBP							
22	CD1C+ dendritic cells	CST3	FCER1A	CD1C					
23	KLRC1+ NCAM1+ NK	GNLY	IL2RB	KLRC1	NCAM1	XCL1	XCL2	GZMK	
24	CD14+ Monocytes (5)	LYZ	VCAN	CD14					
25	TLR7+ plasmacytoid DC	IL3RA	TLR7	CLEC4C					
26	MKI67+ Proliferating	MKI67	HMGB2	TOP2A					
