# Supplementary material for: Association of HBsAg levels with differential gene expression in NK, CD8 T, and memory B cells in treated patients with chronic HBV
Source: JHEP Rep. 2023 Dec 3;6(2):100980. doi: 10.1016/j.jhepr.2023.100980 (PMC10835465; doi:10.1016/j.jhepr.2023.100980)
Supplement: Multimedia component 5 [file mmc5.pdf]

Supplemental Data 2

Clustering of liver cells obtained by fine-needle aspiration

We previously found with bulk RNA sequencing of liver biopsies that genes involved in leukocyte activation, recruitment, and innate responses were positively correlated with liver HBsAg levels (PMID: 34279652). However, it is not clear which cell types were primarily affected by HBsAg and whether these correlations reflect signs of HBsAg-induced immune activation. Therefore, we aimed to identify specific intrahepatic immune subsets in fine-needle aspirates of chronic HBV patients with scRNAseq, to assess which cell types could be affected by HBsAg. First, we identified all immune subsets in the liver (Suppl. Data 2A). Sequencing of FNAs yielded a median of 3,722 high-quality cells (Suppl. Table 7). Ensuring high-quality cell isolation from freshly collected and rapidly processed FNAs is notoriously challenging, due to fragility of especially parenchymal cells, which may partially explain the average 40% loss of cells after quality control and filtering. We identified 29 cell clusters with single-cell clustering of 35,513 intrahepatic cells. Hepatocytes and clusters with less than 500 cells were excluded from downstream analysis. The remaining 19 clusters were found in all patient samples, although one cluster of *CD226*+ *KLRG1*+ NK-like T cells mainly originated from FNA8. This cluster was not equally represented by each patient sample, therefore, gene expression changes in this cluster were not further evaluated. Clusters were annotated according to differentially expressed genes (Suppl. Data 2B-C, Suppl. Table 8). We identified a total of five CD4 T cell clusters, including three clusters of *CCR7*+ *SELL*+ naive CD4 (1-3), *CD40LG*+ CD4, and a *TNFRSF4*+ *CD40LG*+ CD4 T cell cluster. An equal number of CD8 T cell clusters were characterized in the liver: *CCR7*+ *SELL*+ *TCF7*+ naive CD8, *GZMH*+ *TBX21*+ CD8, *CD27*^{low} CD8, *GZMK*+ *CD27*+ CD8, and a liver-resident *CXCR6*+ *CD69*+ CD8 T cell cluster. CD8 T cell clusters had a more than two times higher frequency in the liver vs. blood (35.1% vs. 14.9%) with a lower CD4/CD8 ratio (0.9 vs. 1.6). Less frequent cell clusters were also identified in the liver, such as a *TCL1A*+ naive B cell cluster, a *IGHG*+ *IGHA*+ memory B cell cluster, *CD68*+ macrophages, and one MAIT cell cluster. Lastly, three NK cell clusters were characterized, including a *FCGR3A*+ *NCAM1*^{low} NK cell cluster and two liver-resident NK cell clusters: the *CXCR6*+ *NCAM1*+ NK and *CXCR6*+ *NCAM1*+ *CD160*^{high} NK cell cluster. Separate clustering of FNAs from chronic HBV patients mainly identified distinct CD8 T cell clusters, but also included multiple CD4-, NK-, and B cell subsets, providing a comprehensive overview of intrahepatic and liver-resident immune cell subsets.

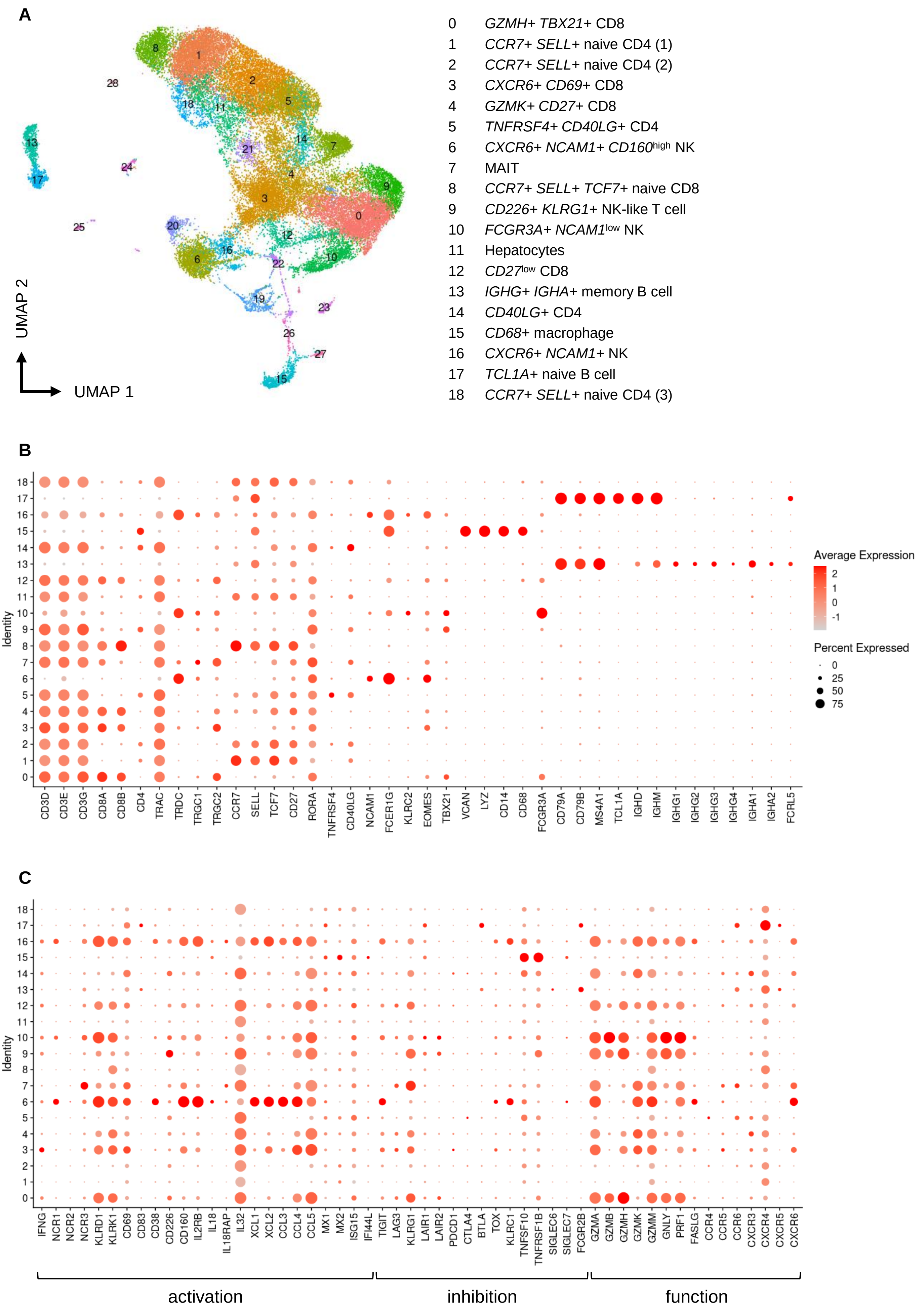

Supplemental Data 2. Clustering of immune cell subsets in liver FNAs (n=35,513). (A) UMAP clustering of 29 cell clusters and annotation of 19 clusters with >500 cells. (B) Differential expressed genes and cell-type specific markers for each cluster. (C) Gene expression markers associated with immune activation, inhibition and functional markers. Color intensity and size of dots represents gene expression level and the percentage of cells expressing the gene of interest, respectively.

Signature genes of clusters in FNAs

No.	Cluster	Signature genes							
0	GZMH+ TBX21+ CD8	CD3D	CD3E	CD3G	CD8A	CD8B	GZMH	TBX21	
1	CCR7+ SELL+ naive CD4 (1)	CD3D	CD3E	CD3G	CD4	CCR7	SELL		
2	CCR7+ SELL+ naive CD4 (2)	CD3D	CD3E	CD3G	CD4	CCR7	SELL		
3	CXCR6+ CD69+ CD8	CD3D	CD3E	CD3G	CD8A	CD8B	CD69	CXCR6	
4	GZMK+ CD27+ CD8	CD3D	CD3E	CD3G	CD8A	CD8B	GZMK	CD27	
5	TNFRSF4+ CD40LG+ CD4	CD3D	CD3E	CD3G	CD4	TNFRSF4	CD40LG		
6	CXCR6+ NCAM1+ CD160high NK	NKG7	NCAM1	CXCR6	CD160	PRF1	IL2RB	FCER1G	TIGIT
7	MAIT	CD3D	CD3E	CD3G	IL7R	RORA	KLRB1		
8	CCR7+ SELL+ TCF7+ naive CD8	CD3D	CD3E	CD3G	CD8A	CD8B	TCF7	CCR7	SELL
9	CD226+ KLRG1+ NK-like T cell	CD3D	CD3E	CD3G	GNLY	PRF1	NKG7	CD226	KLRG1
10	FCGR3A+ NCAM1low NK	GNLY	NKG7	FCGR3A	PRF1				
11	Hepatocytes	ALB	APOC3	APOA2	APOC1				
12	CD27low CD8	CD3D	CD3E	CD3G	CD8A	CD8B			
13	IGHG+ IGHA+ memory B cell	MS4A1	CD79A	CD79B	AIM2	IGHM	IGHG1-4	IGHA1-2	
14	CD40LG+ CD4	CD3D	CD3E	CD3G	CD4	CD40LG			
15	CD68+ macrophage	LYZ	S100A8	S100A9	VCAN	CD68	CD14		
16	CXCR6+ NCAM1+ NK	NKG7	GNLY	IL2RB	PRF1	FCER1G	NCAM1	CXCR6	
17	TCL1A+ naive B cell	MS4A1	CD79A	CD79B	IGHM	IGHD	TCL1A	FCER2	
18	CCR7+ SELL+ naive CD4 (3)	CD3D	CD3E	CD3G	CD4	CCR7	SELL		
